# SpySwitch enables pH- or heat-responsive capture and release for plug-and-display nanoassembly

**DOI:** 10.1038/s41467-022-31193-8

**Published:** 2022-06-28

**Authors:** Susan K. Vester, Rolle Rahikainen, Irsyad N. A. Khairil Anuar, Rory A. Hills, Tiong Kit Tan, Mark Howarth

**Affiliations:** 1 Department of Biochemistry, University of Oxford, South Parks Road, Oxford, OX1 3QU UK; 2 MRC Human Immunology Unit, MRC Weatherall Institute of Molecular Medicine, Radcliffe Department of Medicine, University of Oxford, Oxford, OX3 9DS UK; 3 Present Address: Faculty of Medicine and Health Technology, Tampere University, 33014 Tampere, Finland; 4 Present Address: LiliumX Ltd, WE.306 Westbourne Studios, 242 Acklam Road, London, W10 5JJ UK

**Keywords:** Protein design, Synthetic biology, Protein purification, Nanostructures

## Abstract

Proteins can be empowered via SpyTag for anchoring and nanoassembly, through covalent bonding to SpyCatcher partners. Here we generate a switchable version of SpyCatcher, allowing gentle purification of SpyTagged proteins. We introduce numerous histidines adjacent to SpyTag’s binding site, giving moderate pH-dependent release. After phage-based selection, our final SpySwitch allows purification of SpyTag- and SpyTag003-fusions from bacterial or mammalian culture by capture at neutral pH and release at pH 5, with purity far beyond His-tag methods. SpySwitch is also thermosensitive, capturing at 4 °C and releasing at 37 °C. With flexible choice of eluent, SpySwitch-purified proteins can directly assemble onto multimeric scaffolds. 60-mer multimerization enhances immunogenicity and we use SpySwitch to purify receptor-binding domains from SARS-CoV-2 and 11 other sarbecoviruses. For these receptor-binding domains we determine thermal resilience (for mosaic vaccine development) and cross-recognition by antibodies. Antibody EY6A reacts across all tested sarbecoviruses, towards potential application against new coronavirus pandemic threats.

## Abstract (web summary)

The SpyCatcher-SpyTag system allows protein anchoring and nanoassembly. Here, the authors engineer SpySwitch, a dually switchable Catcher which allows gentle purification of SpyTagged proteins prior to downstream applications such as the assembly of virus-like particles.

## Introduction

Widely applicable and simple technologies help to accelerate progress and democratize biological exploration^1^. Isolation of untagged proteins is an art-form, with painstaking combinations of columns and precipitations^2^. However, it is now expected that a single affinity column should recover low abundance tagged proteins with high purity and activity^3^. There are a wide range of tags for purification, with large tags focusing on enhancing solubility (e.g. maltose binding protein, MBP), and the most widely used small tag being the His_6_-tag^3,4^. Most purification tags add little to the utility of that protein after purification, beyond non-covalent detection. SpyTag (and the latest version SpyTag003) can add substantially to protein utility, because they allow irreversible and site-specific coupling to their SpyCatcher partner. SpyTag reactivity allows simple multiplexing of protein function (e.g. with different fluorophores, enzymes or toxins), stable orientation onto surfaces (beads, cells, chips, hydrogels, cryo-EM grids), and nanoassembly into various oligomeric structures (e.g. for vaccination)^5–10^. Therefore, it would be preferable if the SpyTag served for purification, so that there was no need to add a further tag. We made an initial attempt to establish SpyTag-based purification, through a point mutation in a SpyCatcher scaffold to block reaction (E77A), along with introducing a cysteine for resin anchoring, yielding the protein termed SpyDock^11^. SpyDock allowed high yield purification, but SpyTag was bound so tightly to SpyDock that stringent conditions (2.5 M imidazole) or pH 2 were needed for elution^11^. With no specific route to destabilize SpyDock, these elution conditions would lead to global disruption of the protein, which is likely to perturb any protein to which the SpyTag is fused. Since then we further developed the SpyTag003/SpyCatcher003 pair with 400-fold faster reactivity than SpyTag/SpyCatcher, dependent on an even tighter binding of the peptide to its protein partner^12^. Therefore, it was important to establish gentler elution conditions that would be applicable to both SpyTag-fusions (still being widely used by many groups)^13^ and SpyTag003-fusions (with optimal reactivity) (Tag sequences compared in Supplementary Fig. 1)^12^. There has been previous work to generate switchable Tag/Catcher pairs, focused on tuning covalent reaction, either by redox^14,15^, temperature^16^ or light^17^. We decided to focus initially on pH-switching, as a potent route to tune protein behavior. Various cell-surface proteins have ligand binding modulated by the difference in pH between the cell-surface and endosome^18^. pH-responsiveness has been engineered into protein folds through structure-based, computational and evolutionary approaches, principally based around the introduction of histidines at the interface^19–21^. Surface-exposed histidine has an average pK_a_ of 6.45^22^. Therefore, at pH 7.0 histidine is mainly uncharged, while at pH 6.0 adjacent histidines will often both be protonated and so lead to charge-charge repulsion.

Here we engineer pH-dependence in non-covalent docking of SpyTag and SpyTag003, generating a SpyCatcher-variant named SpySwitch. While studying SpySwitch’s pH sensitivity, we also establish temperature-sensitivity. Thereafter, we harness SpySwitch for efficient purification, including of antigens important to the generation of a vaccine for broad protection against coronaviruses. Sarbecoviruses are a sub-genus of coronaviruses, including SARS, SARS-CoV-2 and a range of bat viruses, where immune cross-reactivity may be feasible^23–25^. The panel of antigens isolated using SpySwitch enables the identification of an antibody with broad recognition across our full panel of sarbecoviruses.

## Results

### Rational engineering of a pH-switchable SpyTag-binder

**Fig. 1:**
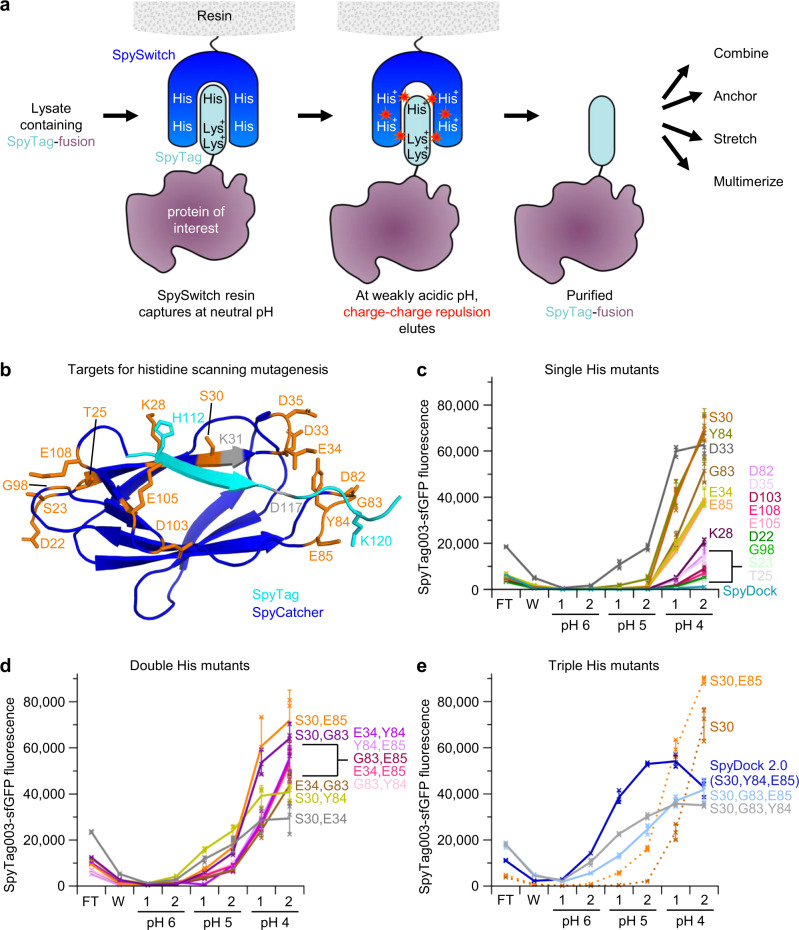
Rational engineering of SpySwitch. **a** Overview of SpySwitch concept. SpyTag fused to a protein of interest interacts with SpySwitch at neutral pH and is eluted at weakly acidic pH through charge-charge repulsion (represented by red stars). **b** Residues at the interface of the SpyTag:SpyCatcher interaction chosen for histidine mutagenesis are marked in orange. SpyCatcher is in dark blue, based on the CnaB2 domain of PDB ID: 2X5P. SpyTag is in cyan, based on PDB ID: 4MLI. Reactive residues K31 and D117 that form an isopeptide bond in SpyTag:SpyCatcher are colored gray. H112 and K120 side-chains on SpyTag (K123 is not resolved in PDB ID: 4MLI) are marked as potential sites of electrostatic repulsion. Certain residues that were selected for mutation in the SpyCatcher003 parent are different to the SpyCatcher grandparent. **c** Single histidine variants of non-reactive SpyCatcher003 were assayed for SpyTag003-sfGFP binding and stepwise pH elution from pH 6.0 to pH 4.0. Each fraction was neutralized before fluorescence detection of SpyTag003-sfGFP. FT, flow-through; W, wash (pH 8.0). **d** Double histidine variants of non-reactive SpyCatcher003 were assayed as in (**c**). **e** Triple histidine mutants were assayed as in (**c**), compared to the best single and double mutant. Individual data points are shown as crosses. The connecting line goes through the mean, with the error bars ± 1 s.d. (*n* = 3). Fluorescence is given in arbitrary units. Source data are provided as a Source data file.

We aimed to establish a pH-switchable SpyCatcher003-based purification system that allows capture of SpyTagged proteins at neutral pH, before eluting under weakly acidic conditions (Fig. 1a). Therefore, we took a rational pH engineering approach through the introduction of histidine residues at the binding interface of SpyTag/SpyCatcher (Fig. 1b). SpySwitch is based on SpyCatcher003 with S49C mutation for site-specific anchoring to the resin and E77A to prevent isopeptide bond formation^11^. Based on the crystal structure, we identified 16 residues on SpyCatcher adjacent to the SpyTag binding site. We individually mutated all 16 of these residues to histidine (Fig. 1b). These single histidine mutants of SpyCatcher003 S49C E77A were coupled to resin and assayed for binding to SpyTag003-superfolder green fluorescent protein (sfGFP) at pH 8.0, with sequential elution at pH 6.0, pH 5.0 and pH 4.0. Our original SpyDock^11^, which shows little pH-sensitivity, was used for comparison. Elution fractions were neutralized, and the fluorescence of sfGFP was measured. S30H, Y84H, G83H, E34H or E85H showed the most promising effect on pH elutability (Fig. 1c) and were chosen to make ten double histidine mutants. D33H gave the best elution at pH 5.0, but more than twice the amount of SpyTag003-sfGFP was lost in the flow-through (Fig. 1c), so we did not pursue this variant. Double mutants S30H + E85H, S30H + G83H or S30H + Y84H showed the most promise, with increased elutability at pH 5.0, but without the compromised binding at pH 8.0 seen for S30H + E34H (Fig. 1d). These encouraging double mutants were combined into three triple mutants and S30H + Y84H + E85H showed best pH elutability at pH 5.0 and 6.0 (Fig. 1e). Therefore, the three mutations S30H, Y84H and E85H were added into SpyCatcher003 S49C E77A, to generate a variant which we termed SpyDock2.0.

### Enhancing pH-responsiveness by phage display selection

**Fig. 2:**
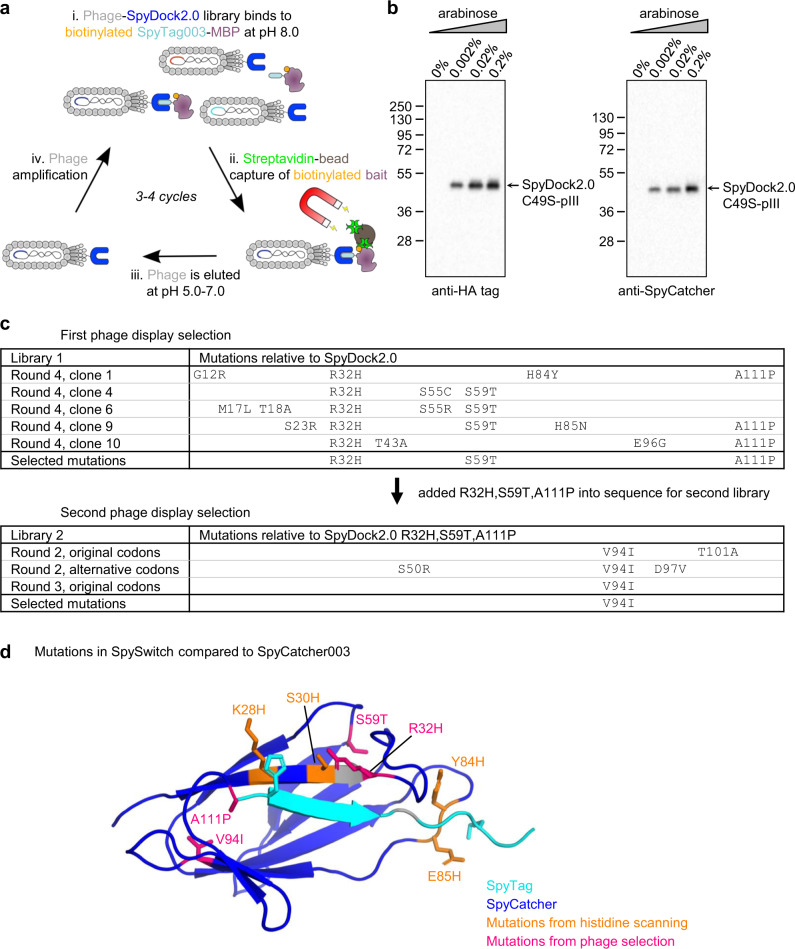
Library selection of SpySwitch. **a** SpySwitch selection strategy. Biotinylated AviTag-SpyTag003-MBP was incubated with a library of M13 phage displaying SpyDock2.0 variants, before pull-down and pH elution. **b** Validation of phage display. Reducing Western blot of phage, induced with increasing arabinose, displaying SpyDock2.0 C49S-pIII, bearing an N-terminal HA tag. The membrane was blotted with anti-HA tag (left gel) or anti-SpyCatcher (right gel). Molecular weight markers represent kDa. **c** Amino acid sequences of phage display libraries. Library 1 is based on SpyDock2.0 C49S. Library 2 is based on successful mutations from library 1. **d** Mutations in SpySwitch. His scanning mutations in orange and phage display mutations in magenta are marked on the structure with side-chains in stick format, based on PDB ID: 2X5P for SpyCatcher (in dark blue) and PDB ID: 4MLI for SpyTag (in cyan). H112 and K120 side-chains on SpyTag (K123 is not resolved in PDB ID: 4MLI) are marked as potential sites of electrostatic repulsion. Source data are provided as a Source data file.

Individually mutating and combining residues has the limitation that cooperativity between different residues is difficult to assess, without expressing and testing an extensive library of variants. To optimize a SpySwitch that shows strong binding at pH 8.0 but high elutability at pH 5.0 and 6.0, we created a phage display library of SpyDock2.0 fused to the pIII C-terminal domain. We included C49S back-mutation to avoid disulfide-mediated dimer formation on phage (Fig. 2a). We optimized display of SpyDock2.0 on phage after inducing with different concentrations of arabinose. Display was validated by Western blot using an antibody against the HA tag on the N-terminus of SpyDock2.0 C49S, as well as with polyclonal anti-SpyCatcher serum (Fig. 2b). Having optimized display, we performed error-prone PCR using Mutazyme II to generate phage library 1 from SpyDock2.0 C49S, with induction in the presence of 0.2% arabinose. We carried out four rounds of selection based on library 1, gradually using more challenging conditions, including changing the elution pH from 5.0 to 6.0. We also introduced free SpyTag003 as a competitor before elution, to select for variants with a low off-rate for the SpyTag003-fused bait. From this screen, we saw repeatedly the three mutations R32H, S59T and A111P (Fig. 2c). We then constructed a second phage display library based on SpyDock2.0 R32H S59T A111P with C49S back-mutation. Library 2 used either the original codons or a template with different codons (104 out of 113 codons changed), to allow for a greater number of amino acids to be accessed through mutation of a single base in the error-prone PCR. Through the rounds of selection, we continued to increase the stringency, including moving the elution buffer from pH 6.5 to 7.0. We also mixed the bait with *Escherichia coli* (*E. coli*) lysate, to favor SpyDock variants with low non-specific binding to the various competing protein targets. Several mutations from the second library were screened, but in the end we only included V94I for increased binding stability. Of the mutations added from phage display selections, R32H and A111P are in direct proximity to SpyTag, while S59T and V94I are distant from the SpyTag binding site (Fig. 2d).

The effect of a histidine mutation is likely to depend on what residues surround it. Therefore, several mutations from the initial rational histidine mutagenesis screen (Fig. 1) were retested in the context of the best phage-derived sequence (SpyDock2.0 R32H S59T V94I A111P). K28H improved pH elutability, especially for SpyTag003, and was added into the final SpySwitch construct (Fig. 2d). The N-terminal region of SpyDock2.0 was further modified by removing the tobacco etch virus (TEV) protease cleavage site and preceding amino acids present in the SpyCatcher003 parent, leaving a His_6_-tag and GSG_2_ linker before the SpySwitch sequence (final sequence in Supplementary Fig. 1).

### Purification of model proteins by pH switch

**Fig. 3:**
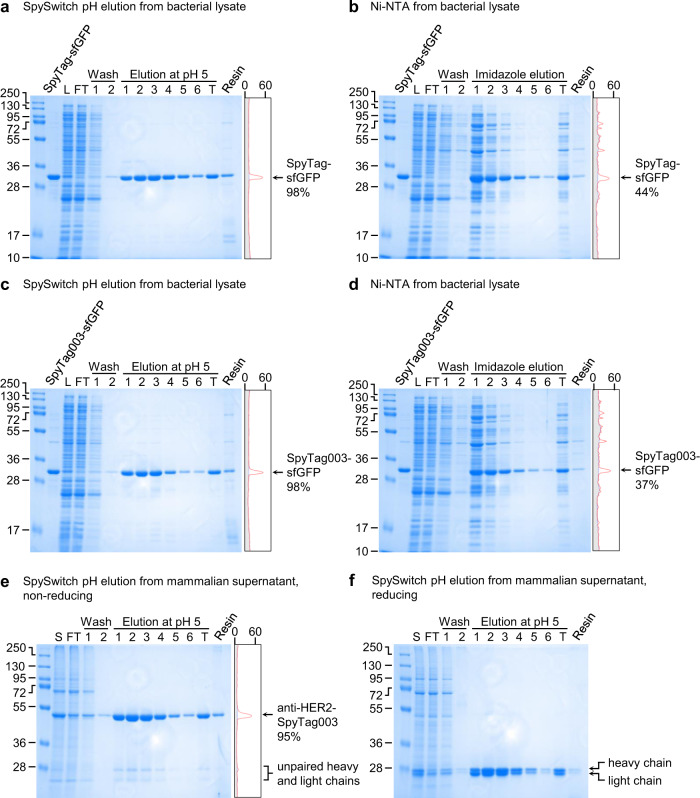
SpySwitch pH-dependent purification. **a** Purification of SpyTag-sfGFP by SpySwitch, eluting with pH 5.0 at 4 °C. Purification of sfGFP bearing an N-terminal SpyTag and a C-terminal His_6_-tag was tested after doping into *E. coli* lysate, assessed by reducing SDS-PAGE/Coomassie. L, doped lysate; FT, flow-through; T, total pooled elution fractions; Resin, protein left on resin following elution. On the right of each gel is the trace from densitometry of lane T (gray, background; orange, bands) plotted by intensity, with percent purity shown. **b** Purification of SpyTag-sfGFP by Ni-NTA after doping into *E. coli* lysate. **c** Purification of SpyTag003-sfGFP by SpySwitch after doping into *E. coli* lysate, eluting with pH 5.0 at 4 °C. **d** Purification of SpyTag003-sfGFP by Ni-NTA after doping into *E. coli* lysate. **e** Purification of anti-HER2 Fab bearing a C-terminal SpyTag003 and His_6_-tag expressed in Expi293F cells using SpySwitch, with elution by pH 5.0 at 4 °C. Purity was assessed by non-reducing SDS-PAGE stained with Coomassie. S, supernatant. The putative unpaired heavy and light chains of the anti-HER2 Fab are marked based on close mobility to the reduced anti-HER2 Fab heavy and light chain in (**f**), and these bands are absent in any other purifications from mammalian culture supernatant. **f** Reducing SDS-PAGE/Coomassie of anti-HER2 bearing SpyTag003 purified by SpySwitch, as shown in (**e**). Molecular weight markers represent kDa. Source data are provided as a Source data file.

Having decided on a final SpySwitch construct, we carefully characterized its performance in purification. We initially tested pH-dependent purification from *E. coli* lysate. Affinity purification of highly expressed proteins is much easier than for low abundance proteins. Therefore, we set up a challenging situation by doping 0.09 mg of N-terminally tagged SpyTag-sfGFP or SpyTag003-sfGFP into cell lysate from 250 mg of *E. coli* wet cell weight^11^. This set-up mimics a protein expressing poorly: low to average recombinant expression was defined as ~1 mg per 250 mg of wet cell weight^26^. This approach also allowed direct comparison between the two tags, since SpyTag-sfGFP and SpyTag003-sfGFP might not show identical levels of expression. Both proteins also contained a His_6_-tag at the C-terminus to allow comparison with the most widely used purification method of nickel-nitrilotriacetic acid (Ni-NTA). For SpySwitch, we eluted with pH 5.0 buffer at 4 °C. For Ni-NTA, we followed a standard procedure of eluting with 200 mM imidazole. We saw that both SpyTag- and SpyTag003-sfGFP purification by SpySwitch occurred at 98% purity (Fig. 3a, c). SpySwitch gave considerably better purity than standard Ni-NTA resin, which gave less than 50% purity using this low input of target protein (Fig. 3b, d). In case of proteins having unusual pH sensitivity, we also showed that substantial SpyTag-sfGFP or SpyTag003-sfGFP could be eluted from SpySwitch at pH 5.5 and some was eluted at pH 6.0 (Supplementary Fig. 2a).

We stored SpySwitch in Tris-phosphate (TP) buffer with 20% (v/v) ethanol at 4 °C, to stop microbial growth. SpySwitch maintained good performance over time, based on purification of SpyTag-sfGFP following SpySwitch storage for 7 months (Supplementary Fig. 2b).

To test SpySwitch purification of proteins expressed in eukaryotic cells, we transiently expressed an anti-HER2 Fab antibody fragment in the human embryonic kidney-based Expi293F cell line (Fig. 3e, f). The construct was tagged with a C-terminal SpyTag003 on the heavy chain, as well as a His_6_-tag. SpySwitch purification from the cell supernatant was efficient, with higher purity observed for SpySwitch (95%) than either SpyDock (89%, using 2.5 M imidazole elution, Supplementary Fig. 3a) or Ni-NTA (88%, using 200 mM imidazole elution, Supplementary Fig. 3b). These different purification methods also gave a product just below 28 kDa, which is likely to be Fab that has not formed a disulfide, based on reducing SDS-PAGE (Fig. 3e, f). SpySwitch purification of anti-HER2 Fab with a C-terminal SpyTag was also effective (Supplementary Fig. 3c). We have not focused on SpyTag002 (Supplementary Fig. 1)^27^, because of the superior reactivity of SpyTag003^12^, but pH elution from SpySwitch was also successful in purifying anti-HER2 Fab-SpyTag002 (Supplementary Fig. 3d).

Overall we have shown that both N- and C-terminally SpyTagged proteins could efficiently be purified by SpySwitch using mild pH elution from bacterial or mammalian expression systems at high purity.

### Biophysical characterization of SpySwitch and its interactions

To get insight into this successful purification behavior, we performed biophysical validation of our SpySwitch construct. We confirmed the molecular identity of SpySwitch by electrospray ionization mass spectrometry (ESI-MS) (Supplementary Fig. 4a). We then used isothermal titration calorimetry (ITC) to find out the binding affinity of SpySwitch. We tested the interaction between monomeric SpySwitch and SpyTag-MBP as well as SpyTag003-MBP, at pH 7.5 and 5 °C (Supplementary Fig. 4b, c). Both SpyTag-MBP and SpyTag003-MBP bound with 1:1 stoichiometry. SpyTag-MBP (K_d_ = 1.1 ± 0.1 μM) (Supplementary Fig. 4b) bound SpySwitch slightly tighter than SpyTag003-MBP (K_d_ = 1.5 ± 0.2 μM) (Supplementary Fig. 4c). No binding between SpySwitch and either SpyTag-MBP or SpyTag003-MBP was observed by ITC at pH 5.0 and 5 °C (Supplementary Fig. 5), suggesting that any interaction present had negligible ΔH or was weaker than the detection limit of ITC.

### SpySwitch is also temperature-responsive

**Fig. 4:**
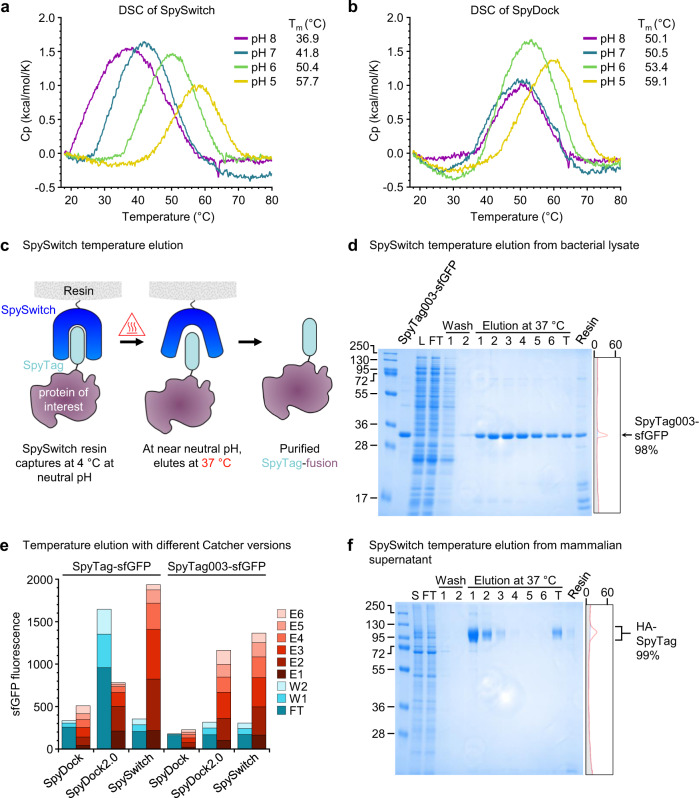
Temperature-dependence in SpySwitch. **a** DSC of SpySwitch at pH 5.0, 6.0, 7.0 or 8.0 in succinate-phosphate-glycine (SPG) buffer. Melting temperature (T_m_) is given at each pH. **b** DSC for SpyDock as in (**a**). **c** Overview of temperature-based elution. SpyTag interacts with SpySwitch at 4 °C and is eluted at 37 °C. **d** Reducing SDS-PAGE performed for SpySwitch temperature elution of SpyTag003-sfGFP from bacterial lysate, with capture at 4 °C and elution at 37 °C (pH 8.0). L, doped lysate; FT, flow-through; T, total pooled elution fractions; Resin, protein left on resin following elution. On the right of the gel is the trace from densitometry of lane T (gray, background; orange, bands) plotted by intensity, with percent purity shown. **e** SpySwitch and SpyDock2.0 but not SpyDock allow temperature elution. SpyTag-sfGFP or SpyTag003-sfGFP was captured by SpyDock, SpyDock2.0 or SpySwitch and eluted by 37 °C incubation at pH 8.0. Fluorescence was determined from flow-through (FT), wash (W), or 37 °C elution (E) fractions (*n* = 1). Fluorescence in arbitrary units was multiplied by the volume of the fraction, to give arbitrary units*mL. **f** SpySwitch temperature elution of HA bearing a C-terminal SpyTag from mammalian Expi293F expression, assessed by reducing SDS-PAGE/Coomassie. S, supernatant; FT, flow-through; T, total pooled elution fractions; Resin, protein left on resin following elution. Molecular weight markers represent kDa. Source data are provided as a Source data file.

It would be beneficial to have an orthogonal method for purification of certain SpyTagged proteins. Important viral antigens can be activated to switch conformation by endosomal pH^28,29^. Therefore, for these proteins pH-dependent elution would not be suitable. We wondered if SpySwitch might allow temperature-dependent elution. Differential scanning calorimetry (DSC) was run on SpySwitch at different pH values (Fig. 4a). At pH 8.0, SpySwitch had a melting temperature of 36.9 °C, suggesting that SpySwitch might release its interaction with SpyTag- or SpyTag003-fusions upon heating up to 37 °C. We also determined how the melting temperature related to pH. Strikingly, SpySwitch’s melting temperature increased by 5–9 °C per pH unit as the pH decreased from 8.0 to 5.0, with the highest melting temperature at pH 5.0 (T_m_ = 57.7 °C) (Fig. 4a). To see if this temperature-dependence was an intrinsic feature of the scaffold, we also tested the unfolding of the parental SpyDock. SpyDock’s melting temperature was well above 37 °C at all pH values and showed less pH-sensitivity than SpySwitch (Fig. 4b). The ΔH and width for each peak is provided in Supplementary Table 1.

We then tested whether we could utilize the temperature-dependence in SpySwitch for purification (Fig. 4c). We doped low levels of SpyTag003-sfGFP into *E. coli* lysate and bound the protein to SpySwitch resin, as previously, in pH 7.5 buffer at 4 °C. We eluted with buffer at 37 °C. This elution buffer had a pH of 8.0, to take advantage of the lower T_m_ of SpySwitch at pH 8.0 than 7.0 (Fig. 4a). The SpyTag003 fusion was purified from the lysate at 98% purity (Fig. 4d). The SpyTag and SpyTag002 fusions were similarly purified by temperature elution from *E. coli* lysate with 98% purity (Supplementary Fig. 6a, c). Temperature elution from SpyDock using the same conditions yielded only a small amount of elution, consistent with the melting temperature of SpyDock (Supplementary Fig. 6b, d). We compared SpySwitch versus SpyDock2.0 or SpyDock for temperature elution of SpyTag-sfGFP and SpyTag003-sfGFP (Fig. 4e). For SpyTag003-sfGFP, temperature elution worked well for SpyDock2.0. However, binding of SpyTag-sfGFP to SpyDock2.0 was severely compromised (Fig. 4e). Very little temperature elution of SpyTag-sfGFP and SpyTag003-sfGFP was seen from SpyDock (Fig. 4e).

To test purification of a pH-sensitive vaccine antigen by SpySwitch^29^, we expressed tagged trimeric influenza hemagglutinin (HA) in mammalian cells and purified from the supernatant, utilizing temperature-switch elution at 37 °C. HA-SpyTag was isolated at 99% purity by temperature elution (Fig. 4f), while HA-SpyTag003 was isolated to 96% purity (Supplementary Fig. 6e). SpySwitch resin recovery was 17.7 mg protein per mL of packed resin when eluted from SpySwitch using pH 5.0 elution. Recovery increased to 20.4 mg when using 37 °C elution (Supplementary Fig. 7). We also showed that the resin could be successfully regenerated. Initially we used SpySwitch resin to purify SpyTag003-sfGFP. Then we regenerated SpySwitch resin with washes of glycine pH 2.0, then 8.0 M urea, before a final wash in 0.1 M sodium hydroxide. We applied the regenerated resin for purification of HA-SpyTag with temperature elution. HA-SpyTag was successfully purified and we were unable to detect any residual SpyTag003-sfGFP, based on Coomassie stain or Western blotting against sfGFP (Supplementary Fig. 8a, b). To assess how the resin withstands multiple rounds of regeneration, we performed purification of SpyTag-sfGFP, regenerated the resin five times, and re-performed purification of SpyTag-sfGFP. No impact on purity of SpyTag-sfGFP was observed, while maximum recovery decreased by 28% from 15.8 mg per mL resin for fresh resin (after 7 months storage in 20% ethanol) to 11.4 mg per mL resin after five regeneration cycles (Supplementary Fig. 8 c, d).

### SpySwitch to purify antigens for broad coronavirus protection

**Fig. 5:**
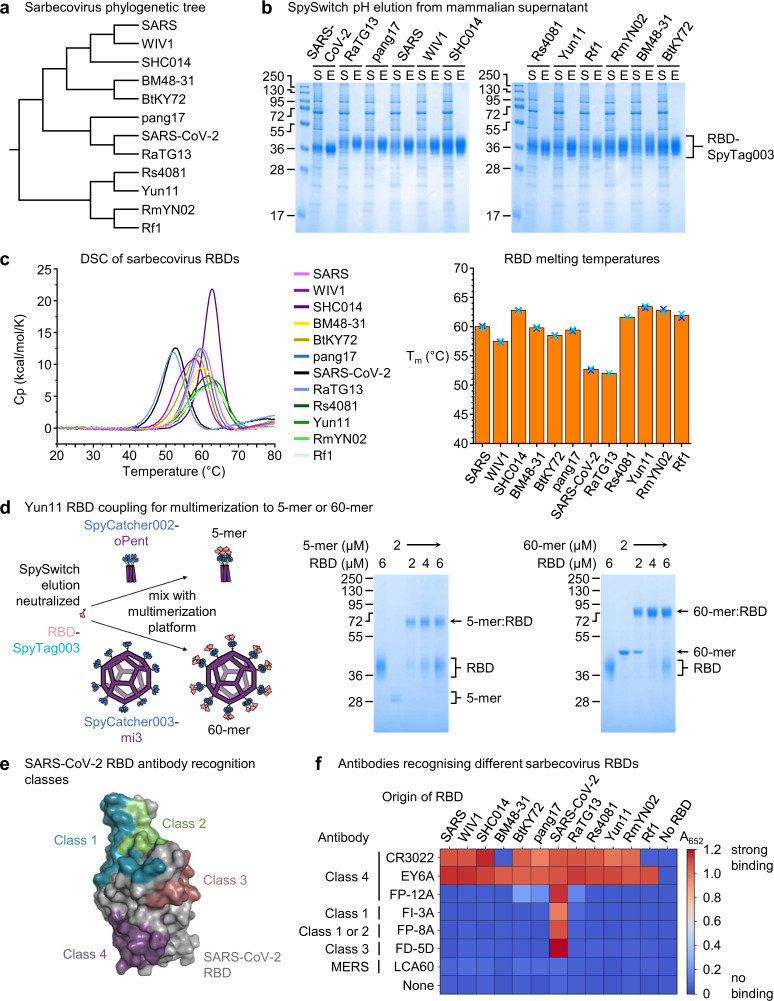
SpySwitch application to a panel of sarbecovirus RBDs. **a** Phylogenetic tree of sarbecovirus RBD constructs. **b** Purification of sarbecovirus RBDs with a C-terminal SpyTag003 using elution by pH 5.0 at 4 °C, assessed by reducing SDS-PAGE/Coomassie. S is supernatant from Expi293F cells and E is the SpySwitch elution. **c** Differential scanning calorimetry of sarbecovirus RBD constructs in PBS pH 7.4. On the right, the melting temperature (T_m_) is plotted for each construct, with the bars in orange representing the mean and the individual values plotted as blue crosses (*n* = 2). **d** Coupling of Yun11 RBD-SpyTag003 to a 5-mer or 60-mer at the indicated concentrations for 16 h at 4 °C in neutralized SpySwitch elution buffer, before reducing SDS-PAGE/Coomassie. **e** SARS-CoV-2 RBD shown with antibody recognition sites class 1 (blue), 2 (green), 3 (bronze) and 4 (purple). **f** Recognition of sarbecovirus RBDs by a panel of antibodies, with mean absorbance at A_652_ from ELISA plotted as a heat map (*n* = 3). Blue represents no binding, while red represents strong binding. Molecular weight markers represent kDa. Source data are provided as a Source data file.

Given the application of SpyCatcher-bearing virus-like particles to facilitate vaccine assembly^30,31^, we applied SpySwitch for purification of vaccine antigens of interest. We recently showed that a mosaic vaccine co-displaying antigens from different sarbecoviruses elicits antibodies that cross-react with the targets across the various viruses^23^. Sarbecoviruses represent the coronavirus sub-genus containing the human pathogens SARS and SARS-CoV-2^24^ (Fig. 5a). We transiently expressed in Expi293F cells twelve sarbecovirus receptor-binding domain (RBD) constructs C-terminally tagged with SpyTag003^23^. We demonstrated efficient purification of each RBD using pH-dependent elution with SpySwitch (Fig. 5b). The band in each case is broad because of heterogeneous N-linked glycosylation^32^.

Thermal stability of antigens is an important characteristic to facilitate robust production and also for future consideration of vaccine cold-chain dependence^33^. We used DSC to validate the integrity of each purified RBD and gain understanding into the different thermal stabilities (Fig. 5c, with ΔH and width for each peak in Supplementary Table 2). DSC showed a single homogeneous peak for each RBD. RaTG13 had a similar T_m_ to SARS-CoV-2 RBD but all the other sarbecovirus RBDs showed substantially higher T_m_ (Fig. 5c).

A purification method would ideally elute the protein of interest in a buffer where it can be easily applied for downstream applications. Buffer-exchange is time-consuming and leads to sample losses^34^. For fusions to SpyTag or SpyTag003, a major application will be reactions with SpyCatcher variants for nanoassembly. For the RBD antigen from the bat coronavirus Yun11, we performed SpySwitch pH-based elution, neutralized, and then mixed with 5-mer (SpyCatcher002-oPent)^11^ or 60-mer (SpyCatcher003-mi3) scaffolds^31^. We saw almost complete depletion of the 5-mer and the 60-mer in the presence of a molar excess of this Yun11 RBD-SpyTag003 sample, consistent with efficient coupling (Fig. 5d).

It is an important question which antibodies are able to cross-react in their recognition of the different sites of RBD, for vaccine efficacy as well as for therapeutic use against new strains of SARS-CoV-2 or potential new zoonotic viruses^23,25^. We tested the RBD recognition by antibodies of class 1 to 4 on SARS-CoV-2 RBD (Fig. 5e) by enzyme-linked immunosorbent assay (ELISA)^35,36^. Recognition is presented as a heat map in Fig. 5f, with mean and error bars in Supplementary Table 3. The antibody CR3022, identified from a SARS patient, had been previously recognized as having broad sarbecovirus reactivity^37^. We found that CR3022 indeed recognized a wide set of RBDs but not RBDs from BM48-31 or Rf1 viruses (Fig. 5f).

In contrast, we found that the antibody EY6A recognized the complete set of sarbecovirus RBDs that we tested (Fig. 5f). EY6A was isolated from a COVID-19 patient^36^. The contact surface of EY6A, binding at the class 4 site on SARS-CoV-2 RBD, has been previously determined^38^ and is shown in Supplementary Fig. 9a, b, along with a sequence alignment of how much this site is conserved through the set of other sarbecovirus RBDs (Supplementary Fig. 9c). The other tested antibodies (FP-12A, FI-3A, FP-8A and FD-5D), derived from COVID-19 patients^36^, only bound strongly to SARS-CoV-2 (Fig. 5f). LCA60 is a control antibody to the Middle East respiratory syndrome-related coronavirus (MERS) spike glycoprotein^39^ and, as expected, showed no recognition of sarbecovirus RBDs (Fig. 5f).

Display of SARS-CoV-2 RBD on the SpyCatcher003-mi3 virus-like particle (VLP) both enhances immunogenicity^23,40^ and enhances the ability of RBD to be detected in vitro by other proteins (ACE2 or antibodies)^36^. We used mi3 for nanoassembly of the RBD panel analyzed in Fig. 5f. We confirmed that each SpySwitch-purified RBD had reacted efficiently with SpyCatcher003-mi3 (Supplementary Fig. 10a). Considering the potential of these proteins for vaccine assembly^23^, we explored the resilience of their folding to freeze-thaw after VLP reaction. Each RBD on mi3 showed no substantial change in its recognition by the pan-reactive conformation-sensitive monoclonal antibody EY6A, even after 5 cycles of freeze-thaw (Supplementary Fig. 10b).

SpySwitch allowed efficient purification of a panel of sarbecovirus RBDs. This panel revealed the high stability of all sarbecovirus RBDs to unfolding. Additionally, we identified a class 4 antibody with recognition all across a wide panel of sarbecoviruses.

## Discussion

We have created a pH- and temperature-responsive protein for efficient purification of proteins fused to SpyTag or SpyTag003, through rational modification and phage library selection. SpySwitch efficiently purifies proteins from bacterial cell lysates as well as from mammalian cell culture supernatant, using either weak acidic elution at pH 5.0 at 4 °C or temperature elution with neutral pH at 37 °C. These mild elution conditions help maintain the folding of the protein of interest, but are also conducive to the good purity we see (>95% from different targets), with harsher conditions more likely to release proteins non-specifically adsorbed to the resin.

SpySwitch is 10 substitutions away from SpyDock. 5 of these mutations are substitution with a histidine (sites 28, 30, 32, 84 and 85), with their close proximity to each other promoting pH-dependence^20^. Therefore, histidine constitutes a large fraction of the surface-exposed residues surrounding the SpyTag binding site (Fig. 2d), which is likely to lead to substantial alteration of the SpySwitch structure as the pH is decreased. Mutations at positions 91 and 103 arose previously during the generation of SpyCatcher003^12^. A111P was identified here from phage display, but was previously found during the selection of SpyTag002^27^. The other two mutations are distant from SpyTag (residue 59) or are buried (residue 94), which would have been hard to predict, so it was important to have performed phage library selection. We wanted to engineer a SpySwitch system that works well for both SpyTag and SpyTag003, enabling application of a single resin. However, this posed a major challenge because SpyTag003 was engineered to have much stronger binding to its Catcher than SpyTag^12^. Initial SpyDock iterations showed excellent capture of SpyTag003-fusions and mediocre capture of SpyTag-fusions, followed by easy elution of SpyTag-fusions and only partial elution of SpyTag003-fusions. However, after testing the large series of histidine-scanning mutants and multiple mutants arising from phage selection for their performance on both SpyTag and SpyTag003, we gradually identified a successful generally-applicable construct. SpySwitch gave solid purification for both SpyTag and SpyTag003, showing similar ~1 µM affinity for each tag. In contrast, SpyCatcher003 interacts with the non-reactive SpyTag003 D117A fused to MBP with ~10 nM K_d_^12^. These data suggest that, by optimizing SpyTag003 elutability at acidic pH, we also lowered SpyTag003’s binding affinity for SpySwitch at neutral pH. SpySwitch’s affinity is comparable to common purification resins (e.g. His_6_/Ni-NTA, Strep-tag II/Strep-Tactin)^41,42^, meaning that it is unlikely that all the SpyTagged molecules in solution will be captured by SpySwitch, but quantitative capture is usually not critical for purification of recombinant proteins.

SpyTag002 has threonine at position 112 instead of the histidine of SpyTag and SpyTag003^12,27^, which might reduce an opportunity for low pH-induced charge-charge repulsion. However, we have seen that pH elution is still successful for SpyTag002. For the sharpest pH-dependent elution profile, we might have introduced further histidines into the Tag, but we avoided that temptation, which would likely compromise the speed of reaction with SpyCatcher003 and would not help the large range of proteins already cloned with SpyTag or SpyTag003^13^. Proteins for secretion in eukaryotes transit through the trans-Golgi network at pH 6.0 as well as secretory vesicles at pH 5.5^43^. Therefore, we anticipate that a large fraction of proteins would tolerate the pH-dependent elution conditions here, but this tolerance will require future testing. Gradual elution does occur from SpySwitch at pH 6.0 (Supplementary Fig. 2a), so this option may be preferred in some cases. Compared to elution with competing ligand, pH elution may allow simple neutralization of the buffer before downstream use, rather than needing any buffer-exchange.

Viral fusion proteins, including influenza hemagglutinin, have evolved to be pH-responsive, so it was important to have another option for elution from SpySwitch. Temperature-sensitive proteins have been a mainstay of genetic screens and there is precedent for rational introduction of temperature-sensitivity by introducing cavities in the protein hydrophobic core, through mutating large buried hydrophobic residues to polar ones^44^. For antibody purification, a temperature-sensitive protein A, eluting at 40 °C, has also been developed to avoid the standard pH 3 step in antibody purification, which can promote deamidation and aggregation^45,46^. Most proteins from mesophilic species are likely to be unaffected by brief exposure to 37 °C, so temperature-based elution should be a widely applicable purification route. Temperature elution has the further important advantage that there is no need to remove the elution buffer, as would often be required for other affinity purification methods, e.g. 200 mM imidazole for Ni-NTA or 10 mM reduced glutathione for glutathione-S-transferase (GST). For vaccine nanoassembly, we previously showed that plug-and-display decoration of virus-like particles is generally applicable for different antigen symmetries^31^. Here we purified monomers (sfGFP, RBDs), a trimer (hemagglutinin) and a tetramer (β-galactosidase) using SpySwitch (Supplementary Fig. 11), so targets bearing multiple tags can still be efficiently eluted. We purified proteins with predicted isoelectric points (pI) ranging from 5.5 to 9.2, suggesting that SpySwitch is broadly applicable (Supplementary Fig. 11).

SpySwitch has the disadvantage that the resin capacity of 20 mg per mL is lower than Ni-NTA and a protein-based capture agent is likely to remain more expensive than an inorganic matrix. However, we found higher purity than Ni-NTA in our model systems, as well as greater flexibility in elution route. Ni-NTA purification also faces challenges such as the His_6_-tag promoting dimerization^47^ and disruption of various metalloproteins^48^. C-tag purification on a nanobody resin has proven a valuable tool for purification of vaccine antigens, with the advantage over SpyTag003 of only a 4 amino acid tag^49^. However, C-tag can only be used at the C-terminus, whereas SpyTag003 is tolerated at either terminus or in loops, while C-tag elution with 2 M MgCl_2_ or 50% propylene glycol^12,50^ can disrupt protein conformation. The CL7-tag has excellent binding affinity but uses pH 3 for elution and is >100 residues^4^. The THETA system uses a temperature-sensitive single-chain Fv fragment for purification of a peptide tag, although includes 50% glycerol in the elution buffer^51^. Split intein-based purification allows removal of tags, but the use of reducing agent to accelerate cleavage and free cysteines in the tag^52^ would be a problem for many complex antigens. Apart from the cysteine for resin anchoring, there are no other cysteines in SpySwitch or any SpyTag version, giving applicability in oxidizing or reducing conditions.

SpySwitch shows tolerance of harsh conditions, including urea and sodium hydroxide, facilitating its regeneration. SpyCatcher and SpyCatcher003 are efficient at refolding^31,53^; we found that SpySwitch could be efficiently regenerated multiple times following a purification run, which will contribute substantially to the cost-effectiveness of SpySwitch resin. SpySwitch also tolerates storage in 20% (v/v) ethanol, facilitating protection from microbial growth and its use over at least 7 months at 4 °C.

In future work, applications of SpySwitch beyond protein purification may be explored. SpySwitch immobilized to resin or magnetic beads may be employed for co-immunoprecipitation, for instance as part of mass spectrometric analysis. SpySwitch may be useful ahead of cryo-electron microscopy structure determination^7^. Beyond isolated proteins, SpySwitch may also be valuable for capture and release of larger SpyTag-linked assemblies, including cells, exosomes and viral vectors^6,10^.

Tag/Catcher technology has been adopted for two clinical trials of vaccine candidates against SARS-CoV-2^54,55^ and is at the heart of the Zoonoses Anticipation Preparedness Initiative’s approach for rapid vaccine construction for future veterinary outbreaks^56^. Similarly, the use of a mosaic vaccine for cross-strain protection^23^ is developing towards the clinic for coronaviruses and other disease challenges. The features we show here are consistent with potential use of SpySwitch as a generic approach to purify candidate vaccine antigens, where the SpyTag will enable vaccine nanoassembly.

In establishing antibodies for broad viral recognition, the class 4 site is a favorable contact surface. EY6A was identified from a SARS-CoV-2 patient and previously shown to cross-react with SARS^38^. We have shown that EY6A achieves good binding across all 12 of the tested sarbecovirus RBDs. This recognition is consistent with the high sequence conservation in the viruses at this site. EY6A was previously shown to bind to full-length SARS-CoV-2 spike and neutralize SARS-CoV-2 infectivity, even though EY6A binds distant from ACE2’s binding site^38^. Future studies will be required to show if that same neutralizing activity is maintained for EY6A against other sarbecoviruses. While this work was in progress, other antibodies have been found that also achieve broad sarbecovirus recognition^57–59^. Overall, SpySwitch illustrates how proteins can become responsive to two distinct features of their environment, while generating a tool that should facilitate the use of Spy technology for empowering protein function.

## Methods

### Plasmids and cloning

Standard PCR methods with Q5 High-Fidelity 2× Master Mix (New England Biolabs) and Gibson assembly were used to perform cloning and site-directed mutagenesis, unless otherwise indicated. All open-reading frames were validated by Sanger sequencing (Source Bioscience). Residue numbers for SpyTag and SpyCatcher variants follow PDB ID: 2X5P^60^.

pDEST14-SpyCatcher2.1 S49C E77A (SpyDock, Supplementary Fig. 1) (GenBank MK637462, Addgene plasmid ID 124618) has been described^11^. pDEST14-SpyCatcher003 S49C E77A variants were derived from pDEST14-SpyCatcher003 (GenBank MN433887, Addgene plasmid ID 133447)^12^, leading to pDEST14-SpyDock2.0 (GenBank ON131073) and then pDEST14-SpySwitch (Supplementary Fig. 1) (GenBank ON131074, Addgene plasmid ID 184225).

pET28a-SpyTag-MBP (GenBank MQ038699, Addgene plasmid ID 35050)^61^, pET28a-SpyTag003-sfGFP (GenBank ON131080, Addgene plasmid ID 133454)^12^, and pET28a-SpyTag003-MBP (GenBank MN433888, Addgene plasmid ID 133450)^12^ have been previously described. pJ404-SpyTag-sfGFP has SpyTag at the N-terminus and His_6_-tag at the C-terminus of sfGFP (GenBank ON131082, Addgene plasmid ID 184226) and was generated by Dr. Karl Brune. pET28a-SpyTag002-sfGFP (GenBank ON131083) was generated by Dr. Matteo Ferla. pET28a-AviTag-SpyTag003-MBP (GenBank ON131081, Addgene plasmid ID 184227) was generated by Dr. Anthony Keeble in the Howarth group. pET28a-SpyCatcher003-mi3 (GenBank MT945417, Addgene 159995) has been described^31^. pDEST14-SpyCatcher002-oPent (GenBank MK637469, Addgene plasmid ID 124664) was previously published^11^. pET28a-His_6_-SpyTag003-β-galactosidase was a kind gift from Juha Huiskonen, University of Helsinki, and contains His_6_-tag, thrombin cleavage site, SpyTag003, GSGESG linker, and *E. coli* β-galactosidase amino acids 1-1024.

pOPIN-anti-HER2 4D5 Fab0.11 light chain (GenBank ON131084) has previously been described^62^. pOPIN-anti-HER2-SpyTag 4D5 Fab0.11 heavy chain (GenBank ON131085) contains SpyTag at the C-terminal end, followed by a His_6_-tag. The heavy chains of pOPIN-anti-HER2-SpyTag002 4D5 Fab0.11 (GenBank ON131075) and pOPIN-anti-HER2-SpyTag003 4D5 Fab0.11 (GenBank ON131076) were cloned using restriction enzymes KpnI-HF and DraIII-HF, with ligation performed using T4 DNA Ligase (all New England Biolabs). pcDNA3.1-H3Vic-SpyTag-His_6_ (GenBank ON131077), encoding the H3 Victoria strain of Influenza HA, was derived from pcDNA3.1-H3Vic-SpyTag003-His_6_ (GenBank MT945422)^31^. Sarbecovirus RBD constructs p3BNC-RBD-His_8_-SpyTag003 have previously been described and were a kind gift from Dr. Alexander Cohen and Prof. Pamela Bjorkman (Caltech)^23^ [SARS-CoV-2 (GenBank ON131086), SARS-CoV (GenBank ON131087), RaTG13-CoV (GenBank ON131088), SHC014-CoV (GenBank ON131089), Rs4081-CoV (GenBank ON131090), pangolin17 (pang17)-CoV (GenBank ON131091), RmYN02-CoV (GenBank ON131092), Rf1-CoV (GenBank ON131093), WIV1-CoV (GenBank ON131094), Yunnan2011 (Yun11)-CoV (GenBank ON131095), BM48-31-CoV (GenBank ON131096), and BtKY72-CoV (GenBank ON131097)].

pBAD-DsbA(ss)-HA tag-SpyDock2.0 C49S-pIII (GenBank ON131078) for phage display was derived from pBAD-DsbA(ss)-SBTI-pIII^63^ with an N-terminally truncated pIII region. Site-directed mutagenesis was performed on the pBAD construct by Gibson assembly to introduce further selected variants. An alternative codon template for SpyDock2.0 R32H C49S S59T A111P (GenBank ON131079) was ordered as a gBlock (Integrated DNA Technologies) and cloned into the phagemid vector. pGEX-2T-GST-BirA was a gift provided by Dr. Chris O’Callaghan (University of Oxford).

Expression plasmids for EY6A^36^, FP-12A, FI-3A (GenBank MT943493 for V_H_ and MT943494 for V_L_)^64^, FP-8A and FD-5D (GenBank MT943497 for V_H_ and MT943498 for V_L_)^64^ based on plasmablasts from either infected or vaccinated donors were a kind gift of Prof. Kuan-Ying Arthur Huang (Chang Gung University). The reverse-transcribed variable heavy (V_H_) and variable light (V_L_) regions were present in plasmids bearing the rest of the human IgG1 heavy chain (AbVec-hIgG1)^65^, human kappa light chain (AbVec1.1-IgKC, Addgene 80796), or human lambda light chain (AbVec1.1-IgLC2, Addgene 99575). The V_H_ and V_L_ segments of LCA60^66^ and CR3022 (GenBank DQ168569 for V_H_ and DQ168570 for V_L_)^67^ were synthesized as cDNA (GeneArt or IDT) and cloned into the heavy and light chain expression plasmids described above.

### Rational selection of histidine mutants

The crystal structure of SpyTag:SpyCatcher (PDB ID: 4MLI)^68^ was visually inspected for potential histidine mutagenesis sites. Interface residues contributing to the binding between SpyTag and SpyCatcher were determined using the InterfaceResidues script in PyMOL 2.0 (Jason Vertrees, https://pymolwiki.org/index.php/InterfaceResidues). Two binding interfaces were identified in SpyCatcher and residues therein were computationally substituted to positively charged histidine residues. These sites were inspected for potential electrostatic charge-charge repulsion with adjacent partially or fully positively charged residues on SpyTag and SpyCatcher within a ~5 Å radius^69^. To target the positively charged N-terminus of SpyTag003, for which no crystal structure is currently available, the N-terminus of SpyTag in PDB ID: 4MLI was computationally extended in PyMOL using the Builder. We added the residues RGV (based on SpyTag003) to the terminal A111 of SpyTag whilst maintaining backbone β-sheet hydrogen bonding to ~3 Å. SpyCatcher residues within a ~5 Å radius to the new terminal R108 side-chain and main-chain amine group were selected for histidine substitution. In total, 16 residues on SpyCatcher003 were individually mutated to histidine residues and assessed for binding to SpyTag003-sfGFP by fluorescence assay. The best single histidine mutants were combined to double histidine mutants, which were in turn combined to triple histidine mutants.

### SDS-PAGE and quantification

SDS-PAGE was performed using an XCell SureLock system (Thermo Fisher) with 10%, 12% or 14% polyacrylamide gels. Samples were mixed with 6× SDS loading buffer [234 mM Tris-HCl pH 6.8, 24% (v/v) glycerol, 120 μM bromophenol blue, 234 mM SDS, supplemented with 60 mM dithiothreitol (DTT) for reduced samples]. Samples were heated at 95 °C for 5 min in a Bio-Rad C1000 thermal cycler. Gels were run in 24 mM Tris base, 192 mM glycine, 3.5 mM SDS at 200 V and stained with Coomassie Brilliant Blue G-250. After destaining in MilliQ water, gels were imaged using a ChemiDoc XRS + imager with ImageLab version 5.2.1, and analyzed with ImageLab version 3.0 (both Bio-Rad). For quantification of protein purity in ImageLab, high sensitivity band detection was performed. Additional bands were added manually where the automatic detection had failed to detect faint bands. 1 mm background subtraction was applied. The percentage purity is quantified as (band intensity of protein of interest)/(sum of the band intensities from all bands in lane)×100. The lane profile was plotted by intensity.

### Bacterial protein expression

pET28a-AviTag-SpyTag003-MBP, pET28a-SpyTag-sfGFP and pET28a-SpyTag003-sfGFP were transformed into chemically competent *E. coli* BL21 (DE3) RIPL (Agilent Technologies). pET28a-SpyTag-MBP, pET28a-SpyTag003-MBP and pET28a-SpyTag003-β-galactosidase were transformed into chemically competent E. cloni EXPRESS BL21(DE3) (Lucigen). pDEST14-SpySwitch (and variants thereof), pDEST14-SpyDock2.0, pDEST14-SpyDock and pDEST14-SpyCatcher002-oPent were transformed into chemically competent *E. coli* C41 (DE3), a gift from Anthony Watts (University of Oxford). Colonies were picked and grown in LB supplemented with 50 μg/mL kanamycin (pET28a) or 100 μg/mL ampicillin (pDEST14) at 37 °C at 200 rpm for 4-16 h. 1 L LB containing 0.8% (w/v) glucose and appropriate antibiotic was inoculated at 1/100 dilution with starter culture and incubated at 37 °C and 200 rpm in ultra-yield baffled flasks (Thomson Instrument Company). At A_600_ 0.5, the cultures were induced with 0.42 mM isopropyl β-D-1-thiogalactopyranoside (IPTG, Fluorochem) and incubated for 4 h at 30 °C and 200 rpm. For pDEST14-SpyCatcher002-oPent, cultures were incubated for 16 h with shaking at 200 rpm at 22 °C after induction. For pET28a-SpyTag003-β-galactosidase, cultures were induced with 0.84 mM IPTG and incubated for 3 h at 37 °C and 200 rpm. For initial SpyCatcher003 S49C E77A histidine variants, a single colony was inoculated into 1 L of autoinduction medium (Formedium) containing 100 μg/mL ampicillin and incubated at 30 °C and 200 rpm for 24 h. Cells were harvested by centrifugation and either directly processed or washed with PBS pH 7.4 (137 mM NaCl, 2.7 mM KCl, 10 mM Na_2_HPO_4_, 2 mM KH_2_PO_4_) and cell pellets were stored at −80 °C until purification.

SpyCatcher003-mi3 was expressed in *E. coli* BL21(DE3) RIPL cells (Agilent) and purified by precipitation with 170 mg ammonium sulfate per mL of cell lysate. The resuspended protein was further purified by size-exclusion chromatography on a HiPrep Sephacryl S-400 HR 16-600 column, which was equilibrated with 25 mM Tris-HCl and 150 mM NaCl at pH 8.5, using an ÄKTA Pure 25 system (GE Healthcare). Fractions were then concentrated with a Vivaspin 20 100 kDa molecular weight cut-off spin concentrator^31^.

### IgG1 expression and purification

ExpiCHO-S cells (Thermo Fisher, A29133) were cultured in an Erlenmeyer flask with vent cap (Corning) at 37 °C with 125 rpm shaking and were transfected with 1 µg/mL of each of the heavy and light chain expression plasmids using the ExpiFectamine CHO Reagent (Thermo Fisher). Transfection mix was prepared in OptiPro Serum Free Media (Thermo Fisher), gently mixed and incubated at 25 °C for 5 min, before being added to the flask. On day 2 after transfection, ExpiCHO Enhancer (Thermo Fisher) and ExpiCHO Feed (Thermo Fisher) were added to the flask and the growth temperature was lowered to 32 °C. On day 5 after transfection, ExpiCHO Feed was added to the flask according to the manufacturer’s instructions. The culture was harvested on day 10 after transfection, by spinning down at 1400 × *g* for 10 min. The supernatant was passed through a 0.22 µm filter (Thermo Fisher). Antibodies were affinity-purified from the supernatant using a MabSelect SuRe (Cytiva) pre-packed column and eluted in 0.2 M sodium citrate pH 3.0, then neutralized with 1.5 M Tris-HCl pH 8.5. The antibodies were desalted using a Zeba Spin Desalting Column (Thermo Fisher) into PBS pH 7.4.

### Mammalian protein expression

Apart from expression of complete IgG1, mammalian expression was carried out in Expi293F cells (Thermo Fisher, A14635). Expi293F cells were maintained in Expi293 Expression Medium (Thermo Fisher) supplemented with 50 U/mL penicillin and 50 µg/mL streptomycin under humidified conditions at 37 °C and 8% (v/v) CO_2_. Cells grown in 125 mL flasks were shaken at 120 rpm, while cells grown in 50 mL mini bioreactor tubes (both Corning) were shaken at 240 rpm. Transfections of Fab, HA and RBD constructs were performed in the absence of antibiotics using the ExpiFectamine 293 Transfection Kit (Thermo Fisher) according to the manufacturer’s instructions. In brief, Expi293F cells were transfected at a density of 3 × 10^6^ cells/mL in 125 mL flasks or 50 mL mini bioreactor tubes with 1 μg total plasmid DNA per mL culture using ExpiFectamine 293 reagent. Approx. 20 hours after transfection, ExpiFectamine 293 Transfection Enhancers 1 and 2 were added. Cell supernatants were collected 5 days after transfection. Other than for Fabs, supernatants were supplemented with cOmplete Mini EDTA-free Protease Inhibitor Cocktail (Roche), centrifuged at 4000 × *g* and 4 °C for 5 min, then passed through a 0.45 μm syringe filter (Thermo Fisher). Supernatants were stored at 4 °C for short periods or frozen at −80 °C before purification.

### Protein purification by Ni-NTA

Purifications were performed at 4 °C. Bacterial cell pellets were thawed and resuspended in Ni-NTA buffer (50 mM Tris-HCl pH 7.8 + 300 mM NaCl) supplemented with cOmplete Mini EDTA-free Protease Inhibitor Cocktail (Roche) and 1 mM phenylmethylsulfonylfluoride (PMSF, Thermo Fisher). For the histidine mutants of SpyDock, incubation with 100 μg/mL lysozyme and 2 U/mL benzonase (both Sigma-Aldrich) was performed for 30 min, rotating at 25 °C. Cells were lysed by sonication on ice at 50% duty cycle for 4 × 1 min with 1 min rest between runs. SpySwitch (and variants thereof), SpyDock2.0 and SpyDock lysates were supplemented with 10 mM 2-mercaptoethanol. Cell lysates were clarified by centrifugation at 30,000 × *g* for 30 min at 4 °C, then incubated with Ni-NTA agarose (Qiagen) for 1 h at 4 °C rolling. Resin was pelleted by centrifugation, then washed with 20 column volumes (CV) of Ni-NTA buffer and Ni-NTA wash buffer (10 mM imidazole in Ni-NTA buffer), supplemented with 10 mM 2-mercaptoethanol for SpySwitch and SpyDock. The resin was transferred to Econo-Pac Chromatography Columns (Bio-Rad) in Ni-NTA wash buffer and washed with a further 20 CV of Ni-NTA wash buffer by gravity flow. Elution was performed with Ni-NTA elution buffer (200 mM imidazole in Ni-NTA buffer) by incubating the resin in at least 4× 1 CV for 5 min. Protein concentrations were determined by A_280_ measurement on a NanoDrop One (Thermo Fisher) using NanoDrop One software version 1.4.2, using extinction coefficients predicted by ExPASy ProtParam^70^. ProtParam was also used for prediction of isoelectric point, based on the protein chain before any glycosylation and with signal sequence cleavage predicted by Signal P 6.0^71^.

SpySwitch, SpyDock2.0 and SpyDock were further purified by size-exclusion chromatography at 4 °C using a HiLoad 16/600 Superdex 75 pg column at 1 mL/min flow rate in 50 mM Tris-HCl, 5 mM ethylenediamine tetraacetic acid (EDTA), 1 mM Tris(2- carboxyethyl) phosphine hydrochloride (TCEP) (pH 8.5 at 25 °C). Where needed, dialysis was performed into appropriate buffers using 3.5 kDa molecular weight cut-off (MWCO) dialysis tubing (Spectrum Labs). Proteins were concentrated using Vivaspin 6 or 20 centrifugal devices with 5 kDa or 10 kDa MWCO (Cytiva). Typical protein yields per L culture were 20-30 mg for SpySwitch, SpyDock2.0 and SpyDock, 30 mg for SpyTag-MBP, 32 mg for SpyTag003-MBP, 48 mg for AviTag-SpyTag003-MBP, 11 mg for SpyTag-sfGFP, and 6 mg for SpyTag003-sfGFP.

### Resin coupling

SpySwitch variants were coupled to SulfoLink Coupling Resin (Thermo Fisher, 20402) according to the manufacturer’s instructions with some modifications. Briefly, the SpySwitch variant was concentrated to at least 20 mg/mL using a Vivaspin 20 5 kDa MWCO centrifugal filter at 4 °C and reduced with 1 mM TCEP in coupling buffer (50 mM Tris-HCl + 5 mM EDTA, pH 8.5) for at least 30 min at 25 °C. Resin coupling was performed at 25 °C throughout, with incubations protected from light. 20 mg reduced SpySwitch variant was added per 1 mL packed SulfoLink Coupling Resin in coupling buffer containing 1 mM TCEP and incubation was performed first for 30 min on an end-to-end rotator, then for a further 30 min without rotation. After washing with coupling buffer, the column was blocked with 50 mM L-cysteine in coupling buffer by incubating for 15 min on an end-to-end rotator, then for a further 30 min without rotation. Washes with 1 M NaCl, then additionally TP buffer (25 mM orthophosphoric acid adjusted to pH 7.0 with Tris base) were performed to remove non-covalently-bound SpySwitch variant. Coupled resin was stored in 20% (v/v) ethanol in TP buffer.

### pH elution assay by fluorescence

To test binding and elution properties of SpyDock variants, 30 μL packed SpyCatcher003 S49C E77A histidine mutant resins were incubated with 50 μg SpyTag003-sfGFP in a total volume of 300 μL. For the histidine mutant screen performed in Fig. 1c–e, 50 mM 4-(2-hydroxyethyl)-1-piperazineethanesulfonic acid (HEPES) pH 8.0 + 150 mM NaCl was used for binding and washing. Samples were incubated for 45 min at 4 °C with end-over-end rotation. The resin was collected by centrifugation at 1,000 g for 5 min at 4 °C, then transferred to wells of a pre-wetted AcroPrep Advance 0.45 μm Supor membrane 96-well plate (Pall Corporation). The flow-through was collected by centrifugation at 500 g and 4 °C for 30 s. The resin was washed four times with 250 μL 50 mM HEPES pH 8.0 + 150 mM NaCl by centrifugation at 500 g and 4 °C for 30 s. For stepwise elution, resins were sequentially incubated in 2 × 50 μL 50 mM 2-(N-morpholino)ethanesulfonic acid (MES) pH 6.0 + 150 mM NaCl; then 50 mM acetic acid/sodium acetate pH 5.0 + 150 mM NaCl; then 50 mM acetic acid/sodium acetate pH 4.0 + 150 mM NaCl for 10 min at 4 °C. Elution fractions were neutralized by addition of 1 M Tris-HCl pH 8.0 to the collector plates (23 μL for pH 6.0, 10 μL for pH 5.0, 20 μL for pH 4.0). 20 μL of each sample was transferred to a black, flat-bottom half area 96-well plate (Corning) and the plate was incubated at 4 °C for 30 min. Fluorescence was then measured using a CLARIOstar plate reader with CLARIOstar software version 5.20 RS (both BMG Labtech) (λ_ex_  =  482  ±  16 nm, λ_em_  =  530  ±  40 nm) at 30 °C. For analysis, the fluorescence from the different pH elution methods was corrected for differences in final volume. Experiments were performed in triplicate and the mean ± 1 standard deviation (s.d.) was calculated.

To test elution at pH 5.5 and 6.0 (Supplementary Fig. 2a), 50 μg SpyTag-sfGFP or SpyTag003-sfGFP was doped in *E. coli* lysate in 50 mM Tris-HCl pH 8.0 + 300 mM NaCl with binding for 1 h at 4 °C, and wash steps carried out in 300 μL 50 mM Tris-HCl pH 8.0 + 300 mM NaCl. Elution was performed by incubating with either 4× 50 μL 50 mM MES pH 6.0 + 150 mM NaCl, 50 mM MES pH 5.5 + 150 mM NaCl, or 50 mM acetic acid/sodium acetate pH 5.0 + 150 mM NaCl for 10 min at 4 °C. Elution fractions were neutralized by addition of 1 M Tris-HCl pH 8.0 to the collector plates (23 μL for pH 6.0, 23 μL for pH 5.5, 10 μL for pH 5.0). Volumes were adjusted to 73 μL with the respective pH buffer that had been pre-neutralized by addition of 1 M Tris-HCl pH 8.0. 30 μL of each sample was transferred to black, flat-bottom half area 96-well plates and incubated at 25 °C for 30 min. Fluorescence was then measured (λ_ex_  =  482  ±  16 nm, λ_em_  =  530  ±  40 nm) using a FLUOstar Omega plate reader with FLUOstar Omega software version 5.10 R2 (both BMG Labtech) at 25 °C.

sfGFP fluorescence data were processed with MARS Data Analysis Software 3.02 R2 and Microsoft Excel 365 version 16.0.13801.21072, before plotting in GraphPad Prism 9.3.1 or Origin2021b.

### Generation of SpySwitch variants by error-prone PCR

Error-prone PCR was performed on the pBAD construct using the GeneMorph II Random Mutagenesis Kit (Agilent, containing Mutazyme II) for SpyCatcher003 S30H E77A Y84H E85H with 0.25 ng template DNA per 25 µL reaction, with the backbone amplified using KOD Hot Start DNA polymerase. DpnI treatment was performed at 37 °C for 1 h, then inactivated at 80 °C for 20 min. The pBAD-DsbA(ss)-HA tag-SpyDock2.0 C49S-pIII libraries were constructed by Gibson assembly using NEBuilder HiFi DNA Assembly Master Mix (New England Biolabs) with 0.2 pmol of both the insert and the backbone. Assembly reactions were purified using the Wizard SV Gel and PCR clean-up system (Promega) and eluted in nuclease-free water. Aliquots of TG1 phage display electrocompetent *E. coli* (Lucigen) were transformed with 250 ng of library DNA. For each library, 8 aliquots of 25 µL each were transformed. Electroporations were performed in 0.2 mm cuvettes with a MicroPulser (both Bio-Rad) using program EC2. Each electroporation was immediately recovered in 1 mL recovery medium (Lucigen), pooled and incubated for 1 h at 37 °C at 200 rpm. Recovered cells were plated onto 4 bioassay dishes (245 mm × 245 mm, Nunc) with LB agar containing 0.8% (w/v) glucose and 100 μg/mL carbenicillin and incubated for 16 h at 30 °C. Cells were scraped, transferred to 2×TY containing 0.8% (w/v) glucose and 100 μg/mL carbenicillin, centrifuged and stored in 2×TY containing 20% (v/v) glycerol at −80 °C. The second library was created using both original and alternative codons to allow screening of a larger number of potential codons.

### Phage production and purification

Varying volumes of 2×TY + 2% (w/v) glucose + 0.2% (v/v) glycerol + 100 μg/mL carbenicillin were inoculated from an overnight starter culture of library *E. coli* TG1 [grown in 2×TY, 2% (w/v) glucose, 100 μg/ml carbenicillin]. Cells were grown at 37 °C and 200 rpm until A_600_ 0.5. Cultures were infected with M13KO7 helper phage (New England Biolabs) at a multiplicity of infection of 20 and incubated for 30 min at 37 °C and 80 rpm, followed by 15 min at 37 °C and 200 rpm. Bacterial cells were pelleted by centrifugation at 3,000 g and 4 °C for 10 min and resuspended in the same volume of 2×TY + L-arabinose at 0.2%, 0.02%, 0.002% or 0% (w/v) + 0.2% (v/v) glycerol + 100 μg/mL carbenicillin, then incubated for 30 min at 18 °C and 200 rpm. 50 μg/mL kanamycin was added and the culture was incubated at 18 °C and 200 rpm for 16 h. Cultures were centrifuged at 4,000 g for 15 min at 4 °C and phage was precipitated from supernatant by incubation with 4% (w/v) poly(ethylene glycol) average molecular weight 8,000 (PEG8000, Thermo Fisher) + 0.5 M NaCl for at least 1 h on ice. The phage pellet was collected by centrifugation at 15,000 g and 4 °C for 45 min and resuspended in PBS pH 7.4, with centrifugation at 15,000 g and 4 °C to remove insoluble material. Phage precipitation was repeated twice, before purified phage were stored in 20% (v/v) glycerol in PBS pH 7.4 at −80 °C. Phage stocks were titered in duplicate by quantitative PCR (qPCR) using forward primer 5′-ACTGATTACGGTGCTGCTATCG-3′ and reverse primer 5′-TATCACCGTCACCGACTTGAGC-3′ with 2× SensiMix (Bioline) master mix, relative to a dilution series of M13KO7 (New England Biolabs). qPCR was performed on a Mx3000P qPCR system (Agilent) and data were analyzed using MxPro qPCR software version 4.10 (Agilent).

### Phage display

AviTag biotinylation of AviTag-SpyTag003-MBP was performed with GST-BirA^72^. 100 μM AviTag-SpyTag003-MBP was incubated with 6.6 μM BirA and 1.5 mM biotin in the presence of 5 mM MgCl_2_ and 1 mM ATP for 1 h at 30 °C and 300 rpm. The concentrations of BirA and ATP were doubled and incubation continued for 1 h at 30 °C and 300 rpm. Excess biotin was removed by dialysis into PBS pH 7.4 in three dialysis steps.

Selection with the first library based on SpyCatcher003 S30H E77A Y84H E85H was performed in four rounds. In the first round, 10^12^ colony forming units (cfu) of phage in Protein LoBind tubes (Eppendorf) were incubated in 500 µL with 0.2 μM biotinylated AviTag-SpyTag003-MBP at 4 °C for 16 h, shaking at 500 rpm in Phage Buffer [25 mM Tris-HCl pH 8.0, 500 mM NaCl, 0.05% (v/v) Tween 20] supplemented with 3% (w/v) bovine serum albumin (BSA). Phage bound to biotinylated AviTag-SpyTag003-MBP were captured by incubating in 150 μL BSA-blocked Dynabeads Biotin Binder (Thermo Fisher) magnetic beads per 500 μL reaction for 90 min at 4 °C and 700 rpm. Five washes were performed with Phage Buffer, then phage was eluted by incubating in 200 μL 50 mM acetic acid/sodium acetate pH 5.0 + 150 mM NaCl at 4 °C for 15 min with shaking at 700 rpm. The supernatant was transferred to a fresh BSA-blocked tube and neutralized with 2 M Tris-HCl pH 8.5. Phage were rescued by infecting 2 mL of TG1 cells at A_600_ of 0.5 and produced using M13KO7 helper phage super-infection as described above. The following rounds were performed with the below changes. In the second round, 2 × 10^11^ cfu phage in 200 µL were incubated with 0.1 μM biotinylated AviTag-SpyTag003-MBP for 90 min at 4 °C and 600 rpm. Bead capture with 50 µL magnetic beads was performed with 60 min shaking at 700 rpm at 4 °C. Five washes were performed at 4 °C with Phage Buffer supplemented with 0.5% (v/v) Tween 20 and five washes with Phage Buffer. Phage were eluted and rescued as above. In the third round, 10^11^ cfu phage in 200 µL was incubated with 0.05 μM biotinylated AviTag-SpyTag003-MBP for 15 min at 4 °C and 600 rpm. Bead capture with 50 µL magnetic beads was performed for 15 min with shaking at 700 rpm. Washes were performed as in round 2. Phage was eluted with 50 mM MES pH 6.0 + 150 mM NaCl with shaking at 700 rpm for 15 min at 4 °C. In the fourth round, 10^11^ cfu phage was incubated with 0.05 μM biotinylated AviTag-SpyTag003-MBP in Phage Buffer supplemented with competing *E. coli* BL21 (DE3) RIPL lysate for 15 min at 4 °C and 600 rpm. After 10 min incubation, 10 μM SpyTag003 peptide (RGVPHIVMVDAYKRYK, 95% purity, Insight Biotechnology) was added. Bead capture with 50 µL magnetic beads was performed for 25 min in the presence of 10 μM SpyTag003. Five washes were performed with Phage Buffer supplemented with 0.5% (v/v) Tween 20 and then five washes with regular Phage Buffer, with mixing at 700 rpm for 90 s each. Phage were eluted by incubation with 50 mM MES pH 6.0 + 150 mM NaCl with shaking at 700 rpm for 5 min at 4 °C.

Selection with the second library based on SpyCatcher003 S30H R32H S59T E77A Y84H E85H A111P was performed in three rounds. In the first round, 10^12^ cfu phage in 500 µL were incubated with 75 nM biotinylated AviTag-SpyTag003-MBP at 4 °C for 16 h with shaking at 500 rpm in Phage Buffer 2 [25 mM Tris-HCl pH 8.0, 300 mM NaCl, 0.05% (v/v) Tween 20] supplemented with 3% (w/v) BSA. Phage bound to biotinylated AviTag-SpyTag003-MBP were captured by incubating in 150 μL Dynabeads Biotin Binder (Thermo Fisher) per 500 μL reaction at 4 °C for 45 min with shaking at 700 rpm. Five washes were performed with Phage Buffer 2 supplemented with 0.5% (v/v) Tween 20, and five washes with Phage Buffer 2, all in the presence of 7.5 μM free SpyTag003 peptide. The phage were eluted by incubating in 200 μL 50 mM MES pH 6.5 + 150 mM NaCl at 4 °C for 5 min with shaking at 700 rpm. The sample was then neutralized with 2 M Tris-HCl pH 8.5. Phage were rescued by infecting 2 mL TG1 cells at A_600_ 0.5. The following rounds were performed with the below changes. In the second round, 2 × 10^11^ cfu phage were incubated with 50 nM biotinylated AviTag-SpyTag003-MBP for 30 min at 4 °C and 650 rpm shaking in the presence of *E. coli* lysate and the reaction was stopped with 5 μM SpyTag003 peptide. Bead capture was performed for 30 min in the presence of 5 μM SpyTag003 peptide. Five washes were performed with Phage Buffer 2 supplemented with 0.5% (v/v) Tween 20 and then five washes with Phage Buffer 2, all in the presence of 5 μM SpyTag003 peptide. Phage were eluted by incubating in 50 mM HEPES pH 7.0 + 150 mM NaCl at 4 °C with shaking at 700 rpm for 5 min. In the third round, 2×10^11^ cfu phage were incubated with 50 nM biotinylated AviTag-SpyTag003-MBP for 15 min at 4 °C and 650 rpm shaking in the presence of *E. coli* lysate. Then the reaction was stopped with 5 μM free SpyTag003 peptide. Bead capture was performed for 30 min in the presence of 5 μM SpyTag003 peptide. Washes were performed as in round 2. Phage were eluted by incubating in 50 mM HEPES pH 7.0 + 150 mM NaCl at 4 °C with shaking at 700 rpm for 2.5 min.

Sanger sequencing of library clones was performed after each panning round for quality control. A final ten clones were sequenced for library 1 after round 4, five of which are shown in Fig. 2c. A further ten clones were sequenced for the original and for the alternative codon template after round 2 and 3 from library 2. Mutations that occurred at least three times were prioritized for investigation.

### Purification by SpySwitch

For bacterial lysate doping, untransformed *E. coli* BL21 (DE3) RIPL were grown in LB medium to A_600_ 0.5 and incubated with 0.42 mM IPTG at 18 °C and 200 rpm for 16 h. Per gram of wet cell weight, 2 mL SpySwitch wash buffer (50 mM Tris-HCl pH 7.5 + 300 mM NaCl) supplemented with cOmplete Mini EDTA-free Protease Inhibitor Cocktail and 1 mM PMSF was added and cells were lysed by sonication on ice at 50% duty cycle for 4 × 1 min, with 1 min rest after each run. The lysate was clarified by centrifugation at 30,000 g for 30 min at 4 °C and adjusted to pH 7.5 with 1 M Tris-HCl pH 8.0. Lysate was used immediately or stored at −80 °C. For doping, 90 μg SpyTag-sfGFP, SpyTag002-sfGFP or SpyTag003-sfGFP in a small volume of 25 mM Tris-HCl pH 8.0 + 150 mM NaCl was added to a final volume of 500 μL RIPL lysate. On the gel analysis, this doped lysate was denoted as L. For purification from mammalian culture supernatant, 10% (v/v) 10× SpySwitch buffer was added. This adjusted supernatant is denoted as S on the gel analysis.

SpySwitch resin was pre-equilibrated with 2 × 10 CV SpySwitch buffer, then incubated with doped lysate or supernatant for 1 h at 4 °C on an end-over-end rotator. Small-scale purifications were performed in Micro Bio-Spin Chromatography Columns (Bio-Rad) by centrifugation at 500 g using 50 μL packed resin for purification from 0.5 mL bacterial lysate doped with SpyTag-sfGFP, SpyTag002-sfGFP or SpyTag003-sfGFP, 100 μL bacterial lysate expressing SpyTag-MBP, 200 μL bacterial lysate expressing SpyTag003-β-galactosidase or 0.5-1 mL mammalian culture supernatant containing anti-HER2 Fabs, HA-SpyTag or HA-SpyTag003. SpyTag003-β-galactosidase was purified with 10 mM DTT included in the lysate and first two washes, because of β-galactosidase’s multiple surface cysteine residues. Large-scale purifications for RBD-SpyTag003 constructs were performed in Econo-Pac Chromatography Columns (Bio-Rad) by gravity-flow using 0.5 mL (for purification from 15 mL mammalian culture supernatant) or 1 mL packed resin (for purification from 22.5 mL mammalian culture supernatant). The column was drained, with the flow-through (FT) collected. The column was washed with 4 × 10 CV SpySwitch buffer (collected as washes 1 and 2). Elution was performed in either of two ways.

Utilizing the pH switch, bound protein was eluted with 6 × 1.5 CV of 50 mM acetic acid/sodium acetate pH 5.0 + 150 mM NaCl at 4 °C, incubating each fraction for 5 min at 4 °C, with the column capped to prevent flow. When the cap is removed, the protein elutes into microcentrifuge tubes containing 0.3 CV 1 M Tris-HCl pH 8.0, to minimize the time spent at acidic pH. This resultant buffer containing the eluted protein is termed neutralized SpySwitch pH elution buffer. Elution was repeated six times. For total elution (denoted as T or E for RBD constructs) as shown on SDS-PAGE, equal amounts of each elution fraction were mixed. To assess maximum capacity of the SpySwitch resin, elution and neutralization volumes were doubled.

Using the temperature switch, all steps were as above except that bound protein was eluted with 6× 1.5 CV of pre-warmed 50 mM HEPES pH 8.0 + 150 mM NaCl at 37 °C, incubating each fraction for 5 min in a 37 °C incubator, before removing the cap to allow flow.

To assess protein remaining on the resin after elution, 50 µL packed resin was resuspended with 50 µL PBS pH 7.4. 10 µL of this slurry was mixed with 4 µL 6× SDS loading buffer, before heating at 95 °C for 5 min in a PCR machine. 7 µL from the supernatant was then loaded onto SDS-PAGE.

Protein concentrations were determined by A_280_ measurement on a NanoDrop One (Thermo Fisher) or by bicinchoninic acid assay (BCA Protein Assay Kit, Thermo Fisher). Yields from 22.5 mL RBD-SpyTag003 supernatant grown in 125 mL flasks and purified with 1 mL packed SpySwitch resin by pH switch were 80-174 mg per L culture.

### Comparison of Ni-NTA or SpyDock purification with SpySwitch

When comparing SpySwitch against Ni-NTA and SpyDock purification, 50 μL of either packed SpySwitch resin, Ni-NTA agarose or SpyDock resin was used. The resin was equilibrated in 2× 10 CV SpySwitch buffer, Ni-NTA buffer or TP buffer. For purification from bacterial lysate, 90 μg SpyTag-sfGFP or SpyTag003-sfGFP was doped into 500 μL *E. coli* lysate prepared in SpySwitch buffer. For purification from mammalian supernatant, 10% (v/v) of 10× SpySwitch buffer, 10× Ni-NTA buffer or 10× TP buffer was added. After incubation with lysate or supernatant for 1 h at 4 °C, the resin was washed with 4× 10 CV SpySwitch buffer, Ni-NTA wash buffer (Ni-NTA buffer for anti-HER2 Fab) or TP buffer. Elution was performed by incubating for 5 min at 4 °C with 6× 1.5 CV 50 mM acetic acid/sodium acetate pH 5.0 + 150 mM NaCl for SpySwitch, Ni-NTA elution buffer for Ni-NTA, or 2.5 M imidazole in TP buffer at pH 7.0 for SpyDock. SpySwitch elution was neutralized with 0.3 CV 1 M Tris-HCl pH 8.0, while 0.3 CV of Ni-NTA elution buffer or 2.5 M imidazole in TP buffer at pH 7.0 was added to Ni-NTA elution or SpyDock elution, respectively, to adjust volumes.

### Temperature elution with different Catcher versions

50 μL packed resin coupled to SpySwitch, SpyDock2.0 or SpyDock was incubated with 90 μg SpyTag-sfGFP or SpyTag003-sfGFP in 500 μL *E. coli* RIPL lysate in SpySwitch buffer for 1 h at 4 °C. The resin was washed with 4× 10 CV SpySwitch buffer and bound protein was eluted with 6× 1.5 CV of pre-warmed 50 mM HEPES pH 8.0 + 150 mM NaCl at 37 °C, incubating each fraction for 5 min at 37 °C, and the volume adjusted with 0.3 CV of buffer. 30 μL of each sample was transferred to a black, flat-bottom half area 96-well plate and fluorescence was measured using a FLUOstar Omega plate reader (BMG Labtech) at 25 °C.

### Regeneration and storage of SpySwitch resin

In total, 50 μL SpySwitch resin was incubated with 900 μg SpyTag003-sfGFP in bacterial lysate for 1 h at 4 °C. Regeneration was performed at 25 °C directly after binding. For regeneration, the resin was equilibrated with 2× 10 CV SpySwitch buffer. In the first step, the resin was incubated with 3× 10 CV of 0.1 M glycine-HCl pH 2.0 for 5 min each and then re-equilibrated with 2× 10 CV SpySwitch wash buffer. In the second step, the resin was incubated with 3× 10 CV of 50 mM Tris, 8 M urea, pH 7.5 for 5 min each, then re-equilibrated with 2× 10 CV SpySwitch wash buffer. Finally, the resin was incubated with 3× 10 CV of 0.1 M NaOH for 1 min each. The resin was re-equilibrated in SpySwitch wash buffer and incubated with 500 μL HA-SpyTag Expi293F supernatant (that had been supplemented with 10% (v/v) 10× SpySwitch buffer) at 4 °C. Washing and temperature elution were performed as described above. Elution fractions were assessed by SDS-PAGE with Coomassie staining or Western blot for the presence of residual SpyTag003-sfGFP, with known amounts of SpyTag003-sfGFP (100 ng, 10 ng, 1 ng and 0.1 ng) added per lane for comparison.

### Western blot

For phage display validation, 3-4×10^11^ cfu of phage were loaded per lane and proteins from SDS-PAGE were transferred onto nitrocellulose membranes by wet transfer in 12 mM Tris base, 96 mM glycine, 20% (v/v) methanol at 30 V for 2 h using a XCell SureLock system with XCell II Blot Module. Membranes were blocked for 16 h at 4 °C in 5% (w/v) skimmed milk in PBST [PBS pH 7.4 + 0.05% (v/v) Tween 20]. Primary antibodies were diluted in 2.5% (w/v) skimmed milk in PBST, with rabbit anti-HA tag (Rockland Immunochemicals, 600-401-384) at 1:3,000 or mouse polyclonal anti-SpyCatcher serum^73^ at 1:300. Incubation was performed for 80 min at 25 °C. Membranes were washed three times in PBST for 5 min at 25 °C. Secondary antibody detection was performed by staining with goat anti-rabbit IgG horseradish peroxidase (HRP) (Thermo Fisher, 65-6120) or goat anti-mouse IgG HRP (Sigma-Aldrich, A4416) at 1:5,000 for 1 h at 25 °C in 2.5% (w/v) skimmed milk in PBST. After four further washes in PBST at 25 °C, membranes were developed by 5 min incubation at 25 °C with SuperSignal West Pico Plus Chemiluminescent Substrate (Thermo Fisher) and imaged using a ChemiDoc XRS + imager.

For sfGFP detection, proteins were transferred onto nitrocellulose membranes by dry transfer using an iBlot 2 gel transfer device (Thermo Fisher) at 20 V for 10 min. The membrane was blocked for 1 h at 25 °C in 5% (w/v) skimmed milk in PBS pH 7.4 and incubated with mouse anti-GFP (Thermo Fisher, MA5-15256, clone GF28R) at 1:2,000 in 2.5% (w/v) skimmed milk in PBST for 17 h at 4 °C. Secondary antibody staining and detection were performed with goat anti-mouse IgG HRP as described above. Uncropped blots are provided as a Source data file.

### Mass spectrometry

SpySwitch was dialyzed into 50 mM Tris-HCl pH 7.5 + 1 mM TCEP and diluted to 10 μM, then acidified at a final concentration of 0.9% (v/v) formic acid. Samples were loaded onto a C4 solid phase extraction cartridge and washed with 0.1% (v/v) formic acid, then eluted with 85% (v/v) acetonitrile and 0.1% (v/v) formic acid in deionized water. Samples were analyzed using an Agilent 6550 Accurate-Mass Quadrupole Time-of-Flight (Q-TOF) mass spectrometer operated in positive ion mode and utilizing a jet-stream electrospray ion source (Mass Spectrometry Research Facility, Department of Chemistry, University of Oxford). Data were analyzed in Mass Hunter Qualitative Analysis software B.07.00 (Agilent) and protein ionization data were deconvoluted using the maximum entropy algorithm. The mass of reduced SpySwitch without N-terminal formylmethionine was predicted using ExPASy ProtParam.

### Carbamidomethylation of SpySwitch and SpyDock

SpySwitch or SpyDock were reduced in 50 mM Tris-HCl pH 8.5, 5 mM EDTA, 1 mM TCEP. Carbamidomethylation was performed by adding a final concentration of 20 mM iodoacetamide and incubating for 30 min at 25 °C in the dark. Subsequently, proteins were dialyzed into PBS pH 7.4 in the dark with three changes of dialysis buffer. For DSC and ITC, SpySwitch and SpyDock were dialyzed into succinate-phosphate-glycine (SPG) buffer (2.86 mM succinic acid, 10 mM Na_2_HPO_4_, 10 mM glycine, 150 mM NaCl) at pH 5.0, pH 6.0, pH 7.0, pH 7.5, and pH 8.0 or 20 mM Na_2_HPO_4_ pH 7.5 + 150 mM NaCl, as appropriate. SPG was chosen because of its ability to buffer over a wide pH range. Samples were centrifuged at 21,130 g and 4 °C for 10-20 min, prior to concentration measurement by A_280_ in triplicate.

### Isothermal titration calorimetry

Experiments were carried out using a MicroCal PEAQ-ITC calorimeter with MicroCal PEAQ-ITC Software version 1.3 (both Malvern) at 5 °C in 20 mM Na_2_HPO_4_ pH 7.5 + 150 mM NaCl or SPG buffer pH 5.0. Before the experiment, proteins were dialyzed in at least two dialysis steps into the respective buffer, with the last dialysis step at least 12 h. 330 μM SpyTag-MBP or SpyTag003-MBP were titrated into 30 μM carbamidomethylated SpySwitch in the cell with 19 injections. Analysis was carried out using a 1:1 binding model with MicroCal PEAQ-ITC Analysis software version 1.1.0.1262. Error estimates represent the uncertainty of the fit to the binding curve, calculated using MicroCal PEAQ-ITC Analysis software version 1.1.0.1262. The molar ratio refers to the concentration of SpyTagged construct divided by the concentration of carbamidomethylated SpySwitch. The data are representative of two experiments.

### Differential scanning calorimetry

DSC experiments were performed using a MicroCal PEAQ-DSC with MicroCal PEAQ-DSC measurement software version 1.53 (both Malvern). 45 μM carbamidomethylated SpySwitch or carbamidomethylated SpyDock in SPG buffer at pH 5.0, 6.0, 7.0 and 8.0 or 27 μM of sarbecovirus RBD constructs in PBS pH 7.4 were analyzed. Scans were performed from 10 °C to 110 °C at 200 °C/h and 3 atm. Data were analyzed using MicroCal PEAQ-DSC analysis software version 1.53. Buffer and baseline subtraction were performed. Thermal transitions were fitted to obtain the enthalpy of unfolding ΔH and the melting temperature T_m_. The full width at half maximum was determined using Origin2021b (OriginLab).

### RBD multimerization

2, 4, or 6 μM Yun11 RBD-SpyTag003 was incubated with 2 μM (monomer concentration) of either SpyCatcher003-mi3 (60-mer) or SpyCatcher002-oPent (5-mer) for 16 h at 4 °C in neutralized SpySwitch pH elution buffer. Samples were mixed with 6× SDS loading buffer, supplemented with 1 mM DTT, incubated at 95 °C for 10 min, and resolved by 12% SDS-PAGE, before Coomassie staining.

### ELISA

25 nM RBD-SpyTag003 (or PBS pH 7.4 for the no RBD control) was incubated with 25 nM (monomer concentration) SpyCatcher003-mi3 at 4 °C for 48 h in neutralized SpySwitch pH elution buffer, before adsorbing on a clear flat-bottom Immuno Nonsterile 96-Well Plate (Thermo Fisher, 442404) through incubation for 16 h at 4 °C. The wells were washed three times with PBS pH 7.4 with 0.1% (v/v) Tween-20, before blocking with 5% (w/v) skim milk in PBS pH 7.4 for 2 h at 25 °C. The wells were washed three times with PBS pH 7.4 with 0.1% (v/v) Tween-20. 50 nM of the specified antibody (CR3022, EY6A, FP-12A, FI-3A, FP-8A, FD-5D, or LCA60) in 5% (w/v) skim milk in PBS pH 7.4 was added and incubated for 1 h at 37 °C. 5% (w/v) skim milk in PBS pH 7.4 was used for the no antibody control. After three washes with PBS pH 7.4 with 0.1% (v/v) Tween-20, the wells were incubated in a 1/1,600 dilution of goat anti-human IgG conjugated with HRP (Sigma-Aldrich, A8667) in 5% (w/v) skim milk in PBS pH 7.4 for 1 h at 25 °C. A final three washes with PBS pH 7.4 with 0.1% (v/v) Tween-20 were performed. 1-Step™ Ultra TMB-ELISA Substrate Solution (Thermo Fisher) was added to the wells and a time-course of A_652_ measurements was recorded using a FLUOstar Omega plate reader (BMG Labtech) at 25 °C. The mean absorbance from triplicates at 6 min is presented as a heat map in Fig. 5f, with the mean and error bars ± 1 s.d. from triplicate measurements presented in Supplementary Table 3. The results are representative of two separate experiments.

### Freeze-thaw assay

2 μM sarbecovirus RBD-SpyTag003 was incubated with 2 μM (monomer concentration) SpyCatcher003-mi3 for 16 h at 4 °C in neutralized SpySwitch pH elution buffer. The specified number of freeze-thaw cycles was performed by freezing coupled RBD-mi3 by placing in a −80 °C freezer for 20 min and then thawing by incubation in a Thermomixer C (Eppendorf) at 25 °C for 15 min. After the specified number of freeze-thaw cycles was complete, the samples were centrifuged for 30 min at 16,000 g and 4 °C. A 1 in 40 dilution of the supernatant was made in neutralized SpySwitch pH elution buffer and adsorbed on Immuno Nonsterile 96-Well Plates (Thermo Fisher, 442404) through incubation for 16 h at 4 °C. PBS pH 7.4 alone was added to the well for the buffer control. The ELISA was performed with 50 nM EY6A as described above, but the 50 µL reaction was stopped after a 2 min incubation in 1-Step™ Ultra TMB-ELISA Substrate Solution (Thermo Fisher) by adding 50 µL 1 M H_2_SO_4_. A_450_ measurements were performed using a FLUOstar Omega plate reader (BMG Labtech) at 25 °C. Individual data points along with mean absorbance values of triplicate measurements are presented, with error bars ± 1 s.d.

### Graphics and sequence analysis

Protein structures were visualized in PyMOL version 2.0.6 (DeLano Scientific), using PDB ID: 2X5P^60^ to represent SpyCatcher and PDB ID: 4MLI^68^ to represent SpyTag. Antibody classes were defined and contact residues were colored on SARS-CoV-2 RBD from PDB ID: 6M0J^74^, following the Barnes classification^35^. The class 1 binding site was based on PDB ID: 7K8M, class 2 on PDB ID: 7K8X, class 3 on PDB ID: 7K8Z, and class 4 on PDB ID: 6W41^35,37^. Where class 1 and class 2 binding sites had some overlap, we used the class 1 coloring. We used PDB ID: 6ZER for the EY6A/SARS-CoV-2 RBD complex, with contact residues on RBD as defined previously^38^. A phylogenetic tree of sarbecovirus RBD sequences was constructed using MEGA X v 11.0.8 software^75^. Multiple sequence alignment used Clustal Omega v 1.2.4^76^.

### Statistics and reproducibility

For representative SDS-PAGE (Fig. 3a–f, Fig. 4d, f, Fig. 5b, d, Supplementary Fig. 2b, Supplementary Fig. 3a, b, Supplementary Fig. 6 a, c, e, Supplementary Fig. 8 a, c, Supplementary Fig. 10a and Supplementary Fig. 11b, c), observations were confirmed at least once with similar or identical conditions. Phage display without arabinose titration was validated by Western blot (Fig. 2b) at least once under similar conditions. Other SDS-PAGE and Western blot (Supplementary Fig. 3c, d, Supplementary Fig. 6b, d and Supplementary Fig. 8b, d) are the results of a single experiment. SpySwitch and SpyDock DSC results (Fig. 4a, b) were confirmed at least once with identical conditions. Assessment of RBD T_m_ (Fig. 5c, right panel) was performed in two separate experiments. The RBD ELISA (Fig. 5f) was performed in triplicate and confirmed at least once with identical conditions. Mass spectrometry results (Supplementary Fig. 4a) were confirmed once with similar conditions. ITC data were confirmed at least once with identical conditions (Supplementary Fig. 4b, c). SpySwitch capacity (Supplementary Fig. 7) was confirmed once under similar conditions. No statistical method was used to predetermine sample size. No data were excluded from the analyses. The experiments were not randomized. The Investigators were not blinded to allocation during experiments and outcome assessment.

### Reporting summary

Further information on research design is available in the Nature Research Reporting Summary linked to this article.

## Supplementary information

Supplementary Information

Reporting Summary

## Data Availability

Amino acid sequences of SpyDock and SpySwitch are available in Supplementary Fig. 1. Sequences of other constructs are available in GenBank as described in the section Plasmids and cloning. Plasmids encoding pDEST14-SpyDock, pDEST14-SpySwitch, pET28a-SpyTag-MBP, pET28a-SpyTag-sfGFP, pET28a-SpyTag003-sfGFP, pET28a-SpyTag003-MBP, pET28a-AviTag-SpyTag003-MBP, pET28a-SpyCatcher003-mi3 and pDEST14-SpyCatcher002-oPent have been deposited in the Addgene repository (https://www.addgene.org/Mark_Howarth/). Further information and requests for resources and reagents should be directed to and will be fulfilled by the lead contact, M.H. Source data are provided with this paper.

## Source data

Source Data
