# Supplementary material for: SpySwitch enables pH- or heat-responsive capture and release for plug-and-display nanoassembly
Source: Nat Commun. 2022 Jun 28;13:3714. doi: 10.1038/s41467-022-31193-8 (PMC9240080; doi:10.1038/s41467-022-31193-8)
Supplement: Supplementary file 1 — Supplementary Information [file 41467_2022_31193_MOESM1_ESM.pdf]

Supplementary Information

SpySwitch enables pH- or heat-responsive capture and release for plug-and-display nanoassembly

Susan K. Vester[1], Rolle Rahikainen[1,2], Irsyad N. A. Khairil Anuar[1,3], Rory A. Hills[1], Tiong Kit Tan[4] and Mark Howarth[1*]

[1]Department of Biochemistry, University of Oxford, South Parks Road, Oxford, OX1 3QU, UK. [2]Current address: Faculty of Medicine and Health Technology, Tampere University, 33014, Tampere, Finland. [3]Current address: LiliumX Ltd, WE.306 Westbourne Studios, 242 Acklam Road, London W10 5JJ, UK. [4]MRC Human Immunology Unit, MRC Weatherall Institute of Molecular Medicine, Radcliffe Department of Medicine, University of Oxford, Oxford, OX3 9DS, UK.

*Corresponding author

```
                                        1        10        20        30        40
SpyDock          MSYYHHHHHHAMVTTLSGLSGEQGPSGDMTTEEDSATHIKFSKRDEDGRELAGA
SpySwitch  MSYYHHHHHHGSGGSGAMVTTLSGLSGEQGPSGDMTTEEDSATHIHFHKHDEDGRELAGA
                       50        60        70        80        90       100
SpyDock    TMELRDCSGKTISTWISDGHVKDFYLYPGKYTFVATAAPDGYEVATPITFTVNEDGQVTV
SpySwitch  TMELRDCSGKTISTWITDGHVKDFYLYPGKYTFVATAAPDGHHVATPIEFTINEDGQVTV
                  110
SpyDock    NGEATEGDAHTGSSGS*
SpySwitch  DGEATEGDPHTGSSGS*
```

Mutations in SpySwitch

Mutations in SpyCatcher003 not present in SpyDock

Tag and linker sequence

```
             110       120
SpyTag     ---AHIVMVDAYKPTK
SpyTag002  --VPTIVMVDAYKRYK
SpyTag003  RGVPHIVMVDAYKRYK
```

Supplementary Fig. 1 SpySwitch sequence and mutation sites. Amino acid sequence alignment of SpyDock and SpySwitch, as well as SpyTag, SpyTag002 and SpyTag003, numbered according to PDB ID: 2X5P. Residues shown in orange were introduced to generate SpySwitch. Residues in green show mutations present in SpyCatcher003, but not in SpyDock. His_6-tag and linker sequence are shown in gray. * = stop codon.

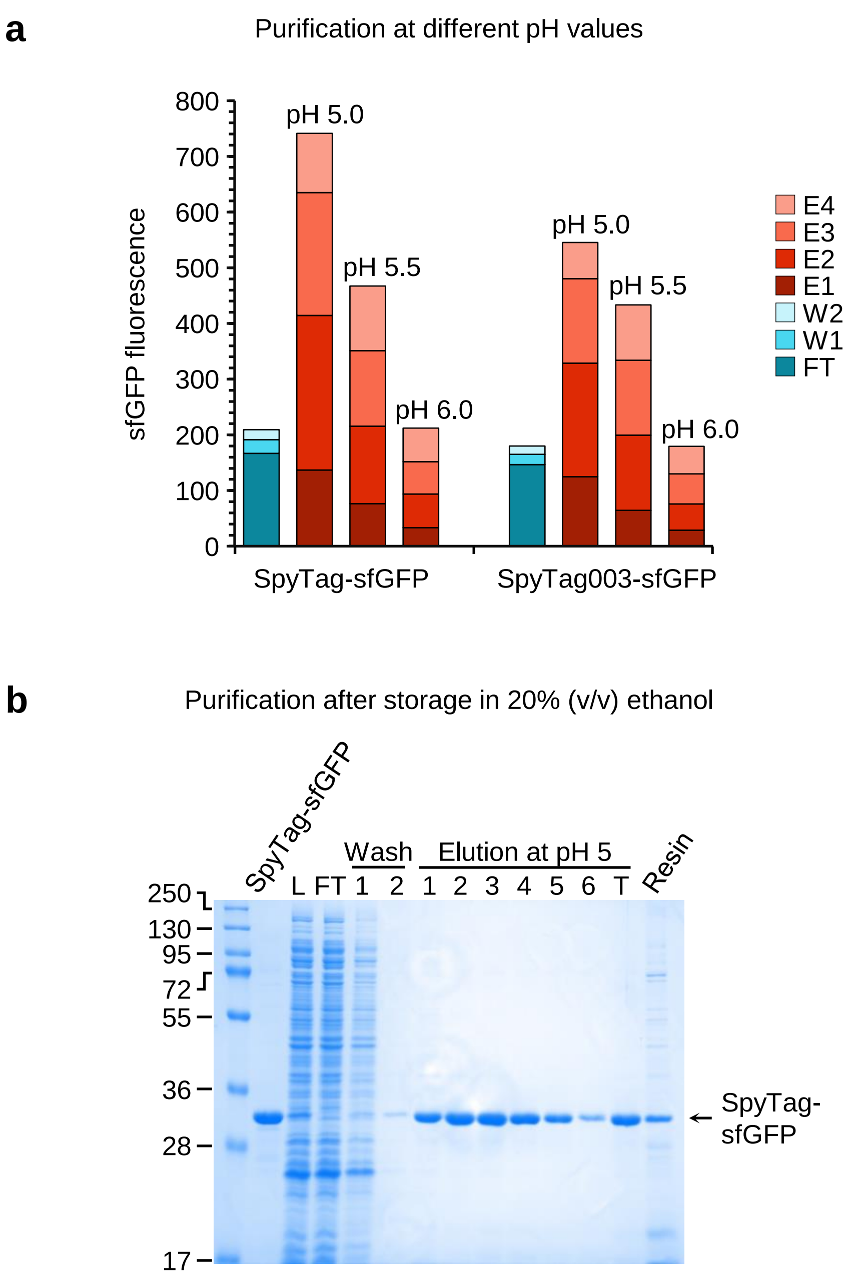

Supplementary Fig. 2 SpySwitch resin can be used for gentler elution and is stable to storage. (**a**) SpySwitch purification at pH values closer to neutral. SpySwitch was used for purification of SpyTag-sfGFP or SpyTag003-sfGFP doped into *E. coli* lysate, with elution at either pH 5.0, pH 5.5 or pH 6.0. After neutralization, fluorescence was determined from the flow-through (FT), wash (W, pH 8.0), or elution (E) fractions (n = 1). Fluorescence in arbitrary units was multiplied by the volume of the fraction, to give arbitrary units*mL. (**b**) SpySwitch resin was stored in 20% (v/v) ethanol in TP buffer pH 7.0 at 4 °C for 7 months, then used for purification of SpyTag-sfGFP doped into *E. coli* lysate. Reducing SDS-PAGE/Coomassie results are shown. L, doped lysate; FT, flow-through; T, total pooled elution fractions; Resin, protein left on resin following elution. Molecular weight markers represent kDa. Source data are provided as a Source data file.

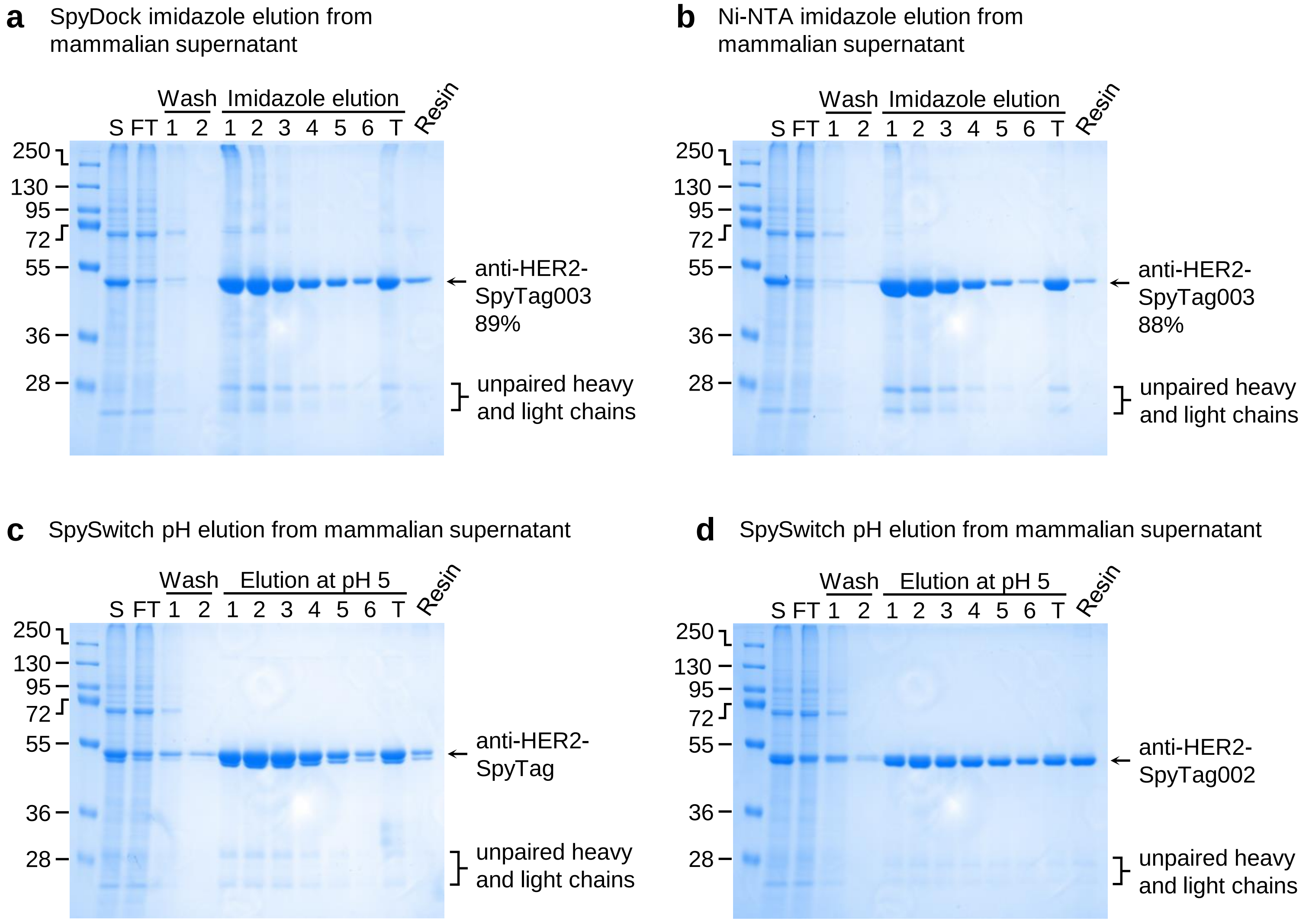

Supplementary Fig. 3 Purification of anti-HER2 Fabs. Purification of anti-HER2 Fab bearing a C-terminal SpyTag003 and His_6-tag expressed in Expi293F cells using (**a**) SpyDock with elution in 2.5 M imidazole in TP buffer pH 7.0 or (**b**) Ni-NTA with elution in 200 mM imidazole in Ni-NTA binding buffer. (**c**) Purification of anti-HER2 Fab bearing a SpyTag and His_6-tag expressed in Expi293F cells using SpySwitch, with elution by pH 5.0 at 4 °C. (**d**) As in (c) bearing SpyTag002. Samples were analyzed by non-reducing SDS-PAGE with Coomassie staining. % purity in (a) and (b) was calculated by densitometry. S, Expi293F supernatant; FT, flow-through; T, total pooled elution fractions; Resin, protein left on resin following elution. The putative heavy and light chains of the anti-HER2 Fab are marked, based on close mobility to the reduced anti-HER2 Fab heavy and light chain shown in Figure 3f, and these bands are absent in any other purifications from mammalian culture supernatant. Molecular weight markers represent kDa. Source data are provided as a Source data file.

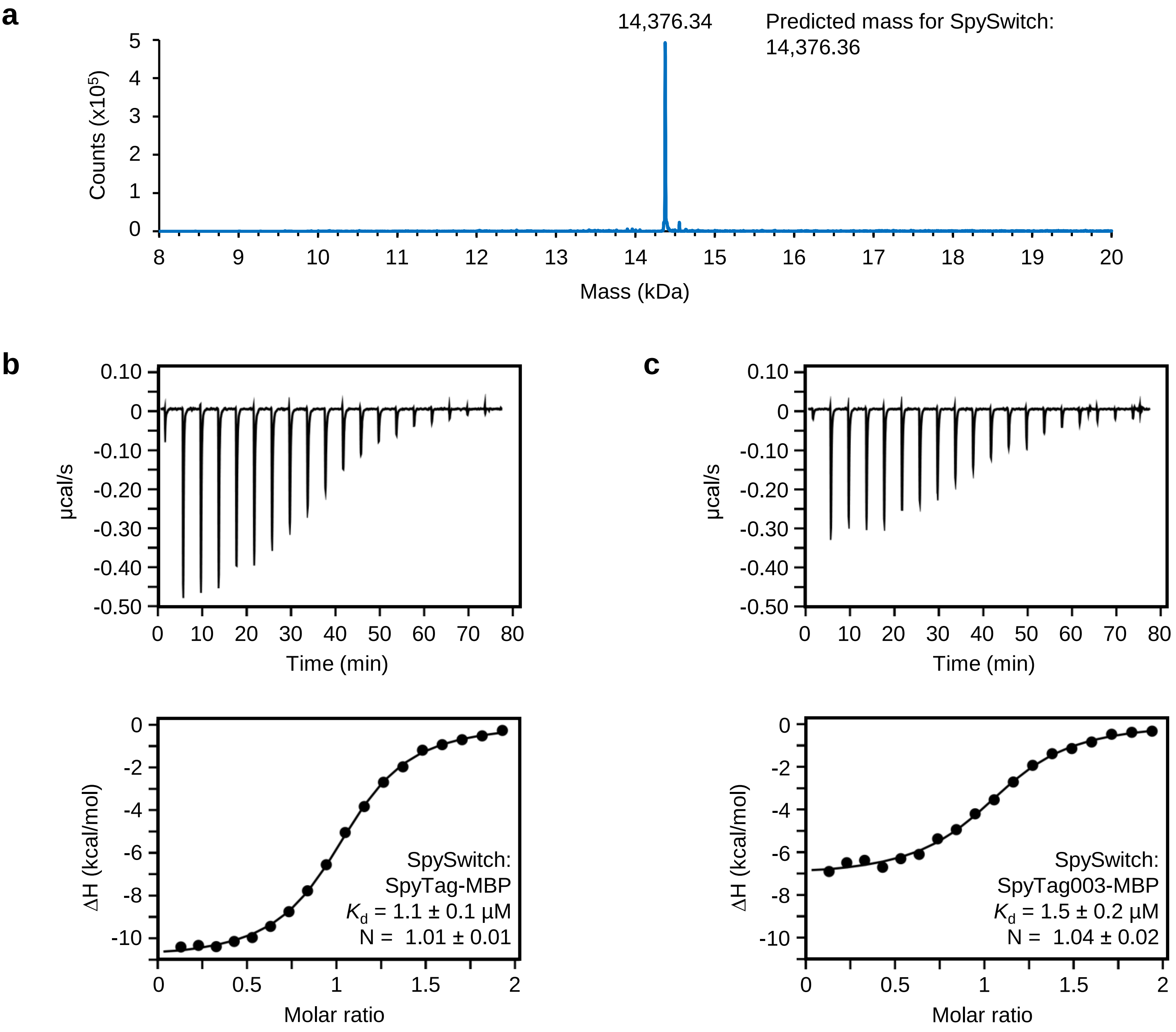

Supplementary Fig. 4 Biophysical characterization of SpySwitch. (**a**) ESI-MS of SpySwitch, showing observed and predicted mass. (**b**) Isothermal titration calorimetry for affinity of SpySwitch to SpyTag-MBP at pH 7.5 and 5 °C. Error estimates represent the uncertainty of the fit. Data are representative of two experiments. (**c**) Isothermal titration calorimetry for affinity of SpySwitch to SpyTag003-MBP, measured as in (b). Source data are provided as a Source data file.

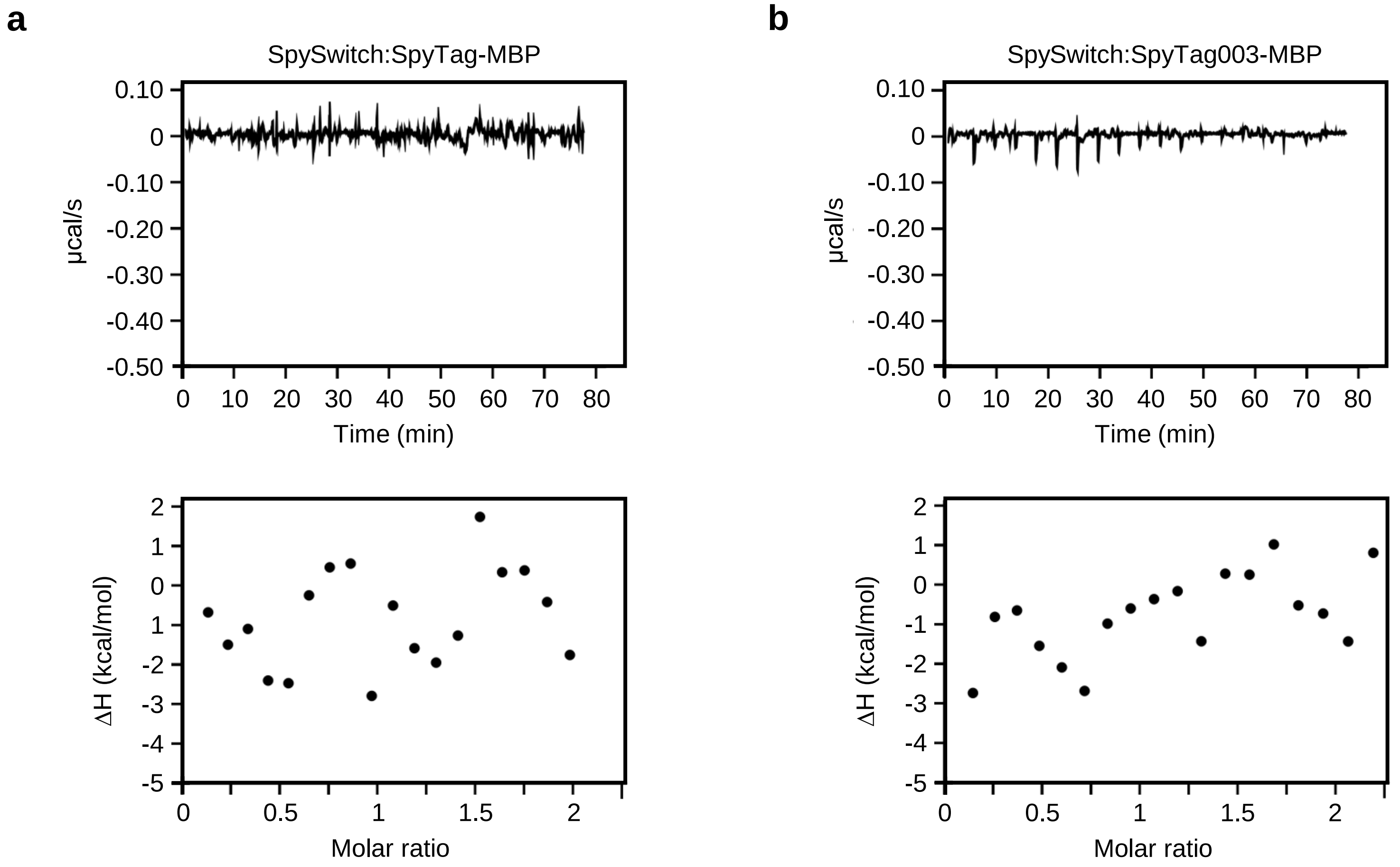

Supplementary Fig. 5 Isothermal titration calorimetry at pH 5.0. ITC showed undetectable interaction of SpySwitch to (**a**) SpyTag-MBP or (**b**) SpyTag003-MBP at pH 5.0 and 5 °C. Source data are provided as a Source data file.

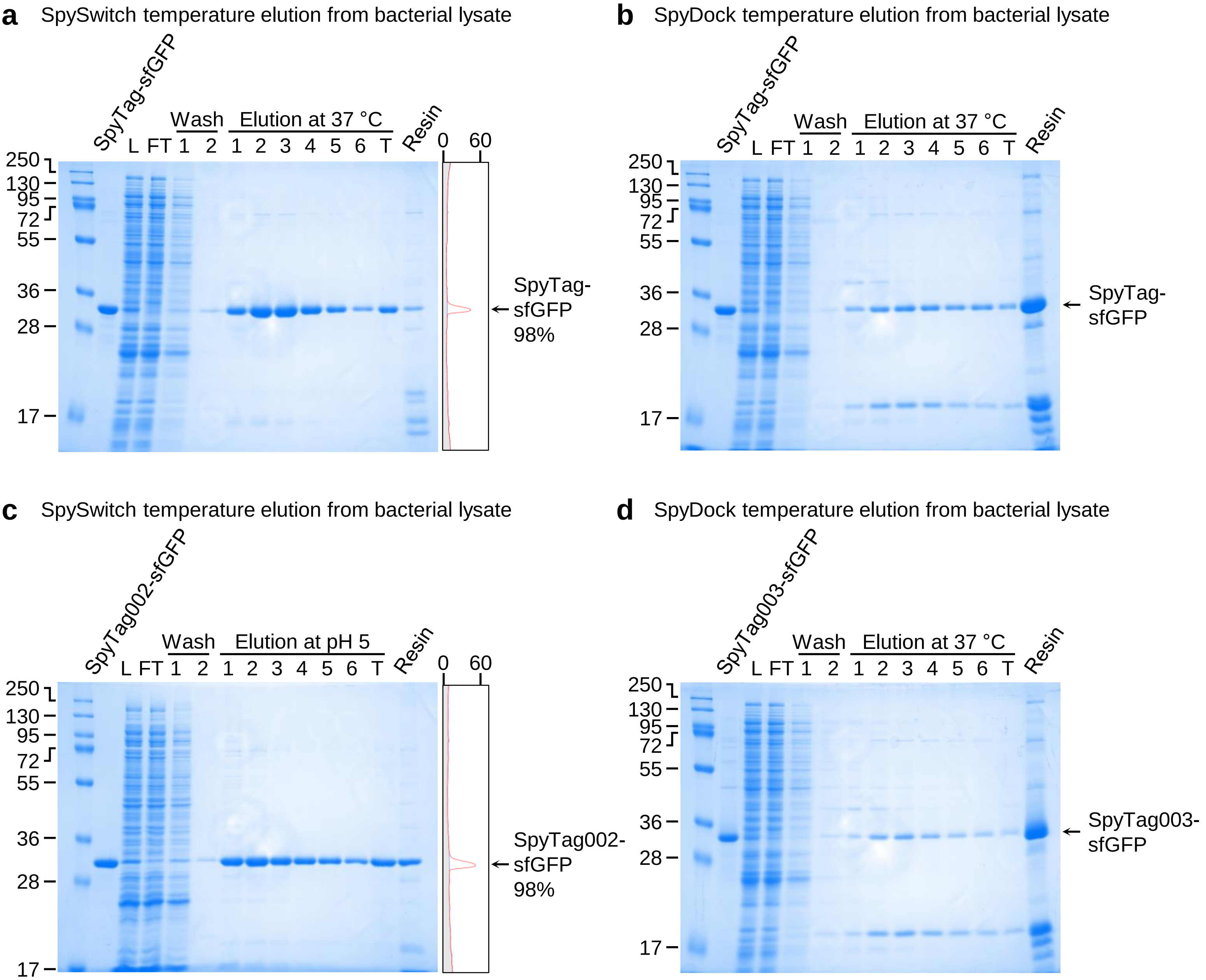

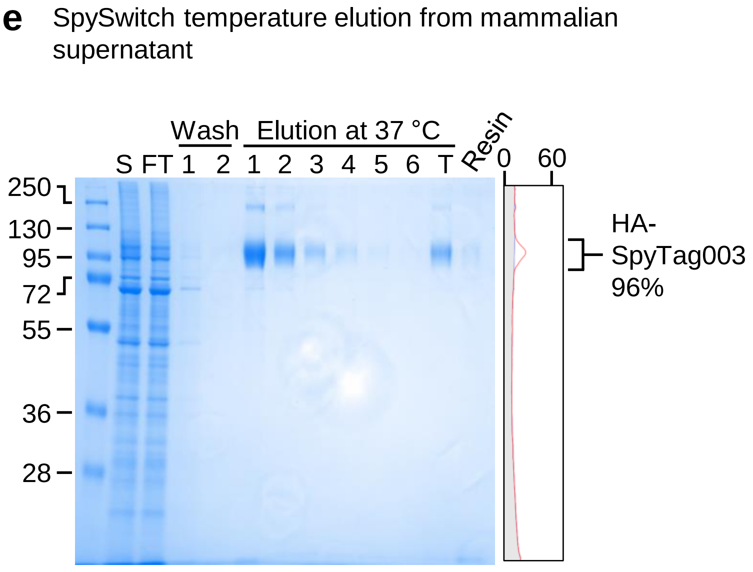

Supplementary Fig. 6 SpySwitch purification from bacterial and mammalian expression systems. (**a**) SpySwitch temperature elution of SpyTag-sfGFP from bacterial lysate, with capture at 4 °C and elution at 37 °C. L, doped lysate; FT, flow-through; T, total pooled elution fractions; Resin, protein left on resin following elution. (**b**) SpyDock temperature elution of SpyTag-sfGFP as in (a). (**c**) SpySwitch temperature elution of SpyTag002-sfGFP as in (a). (**d**) SpyDock temperature elution of SpyTag003-sfGFP as in (a). (**e**) SpySwitch temperature elution of HA-SpyTag003 from mammalian Expi293F expression, with capture at 4 °C and elution at 37 °C. Samples were analyzed by reducing SDS-PAGE with Coomassie staining. % purity in (a), (c) and (e) was calculated by densitometry. S, supernatant. Molecular weight markers represent kDa. Source data are provided as a Source data file.

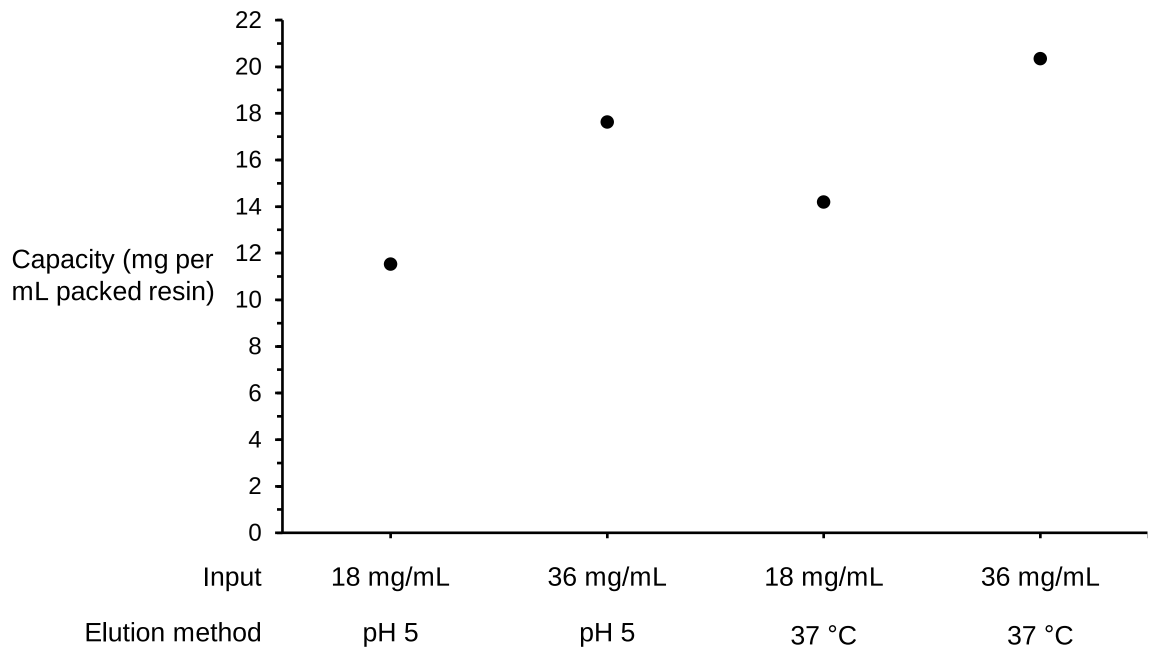

Supplementary Fig. 7 SpySwitch resin capacity. SpySwitch resin capacity was determined by doping SpyTag-sfGFP into bacterial lysate at 18 mg or 36 mg per mL packed resin and purifying by pH switch at pH 5.0 or temperature switch at 37 °C. Protein concentration was measured by A_{280} after elution and the yield was calculated. Source data are provided as a Source data file.

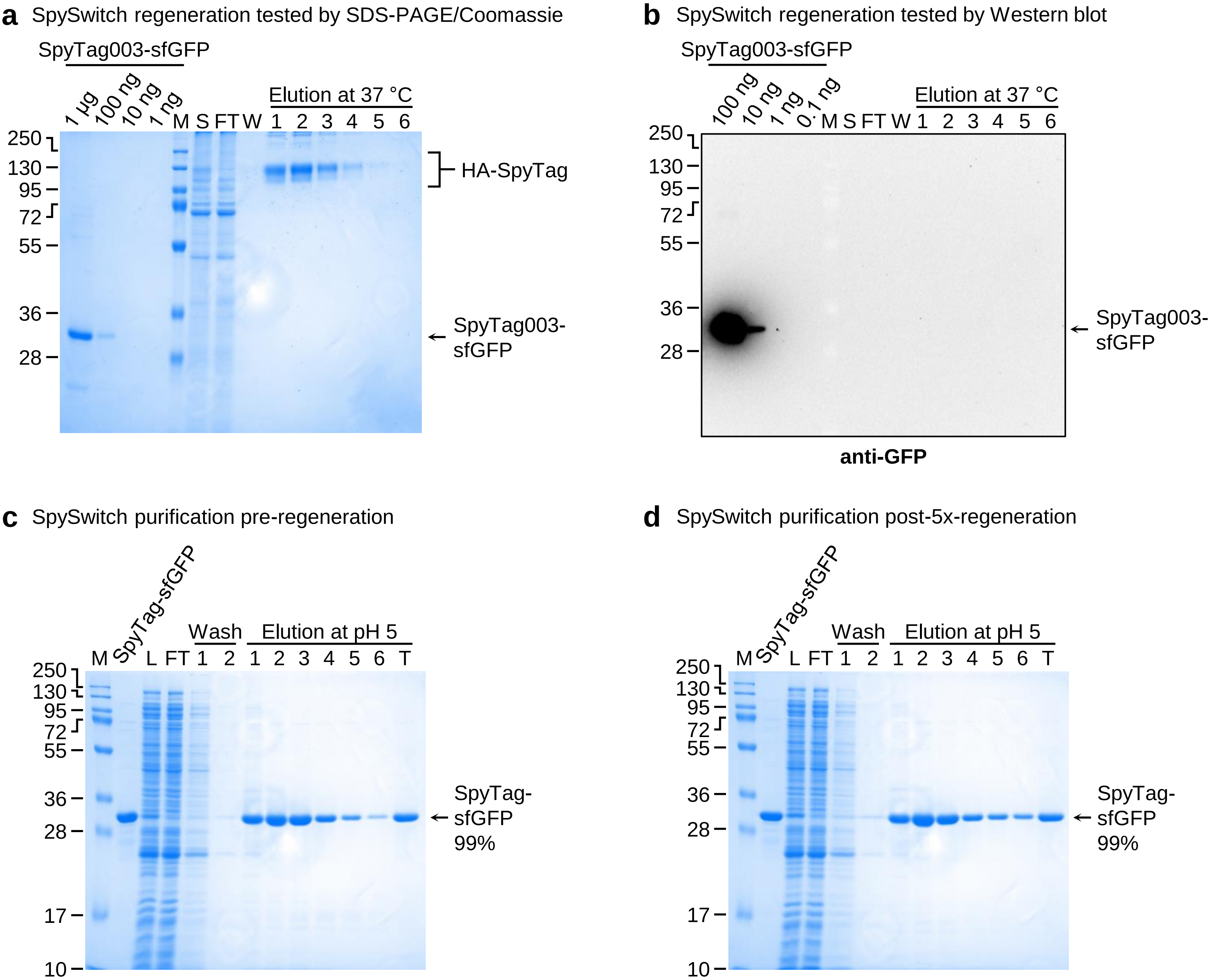

Supplementary Fig. 8 SpySwitch resin can be regenerated multiple times. (**a**) SpyTag003-sfGFP was bound to SpySwitch resin, before regeneration by sequential washes with 0.1 M glycine pH 2.0, then 50 mM Tris-HCl pH 7.5 + 8 M urea, and finally 0.1 M NaOH. Regenerated SpySwitch resin was used for purification of HA-SpyTag from Expi293F cells with temperature elution at 37 °C. SpyTag003-sfGFP was loaded in varying amounts as a standard to allow for calibration. Samples were analyzed by reducing SDS-PAGE with Coomassie staining. M, molecular weight markers; S, Expi293F supernatant; FT, flow-through; W, wash. (**b**) Western blot analysis of regeneration, performed as in (a), staining with anti-GFP. (**c**) SpySwitch purification from fresh resin. SpyTag-sfGFP was doped into *E. coli* lysate and fresh SpySwitch resin was used for purification by pH elution. Samples were analyzed by reducing SDS-PAGE with Coomassie staining. % purity was calculated by densitometry. L, doped lysate; FT, flow-through; T, total pooled elution fractions. (**d**) SpySwitch purification from resin that had been regenerated 5 times. Purification was conducted as in (c) after five rounds of regeneration. Molecular weight markers represent kDa. Source data are provided as a Source data file.

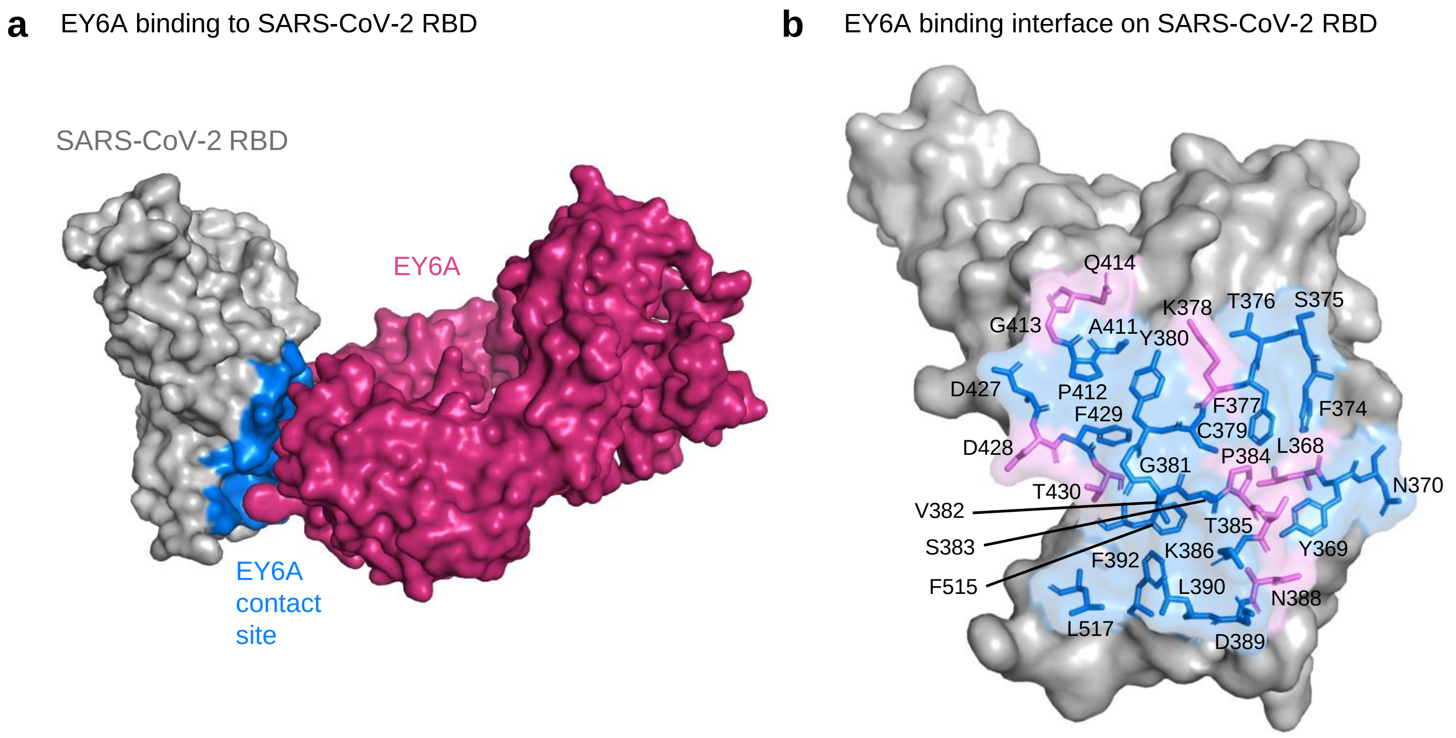

c

```
             320       330       340       350       360       370       380       390       400       410       420
SARS       RVVPSGDVVRFPNITNLCPFGEVFNATKFPSVYAWERKKISNCVADYSVLYNS-TFFSTFKCYGVSATKLNDLCFSNVYADSFVVKGDDVRQIAPGQTGVIADYNYKLPDDF
WIV1       RVAPSKEVVRFPNITNLCPFGEVFNATTFPSVYAWERKRISNCVADYSVLYNS-TSFSTFKCYGVSATKLNDLCFSNVYADSFVVKGDDVRQIAPGQTGVIADYNYKLPDDF
SHC014     RVAPSKEVVRFPNITNLCPFGEVFNATTFPSVYAWERKRISNCVADYSVLYNS-TSFSTFKCYGVSATKLNDLCFSNVYADSFVVKGDDVRQIAPGQTGVIADYNYKLPDDF
BM48-31    RVTPTTEVVRFPNITQLCPFNEVFNITSFPSVYAWERMRITNCVADYSVLYNSSASFSTFQCYGVSPTKLNDLCFSSVYADYFVVKGDDVRQIAPAQTGVIADYNYKLPDDF
BtKY72     RVSPSTEVVRFPNITNLCPFGQVFNASNFPSVYAWERLRISDCVADYAVLYNSSSSFSTFKCYGVSPTKLNDLCFSSVYADYFVVKGDDVRQIAPAQTGVIADYNYKLPDDF
pang17     RVQPTISIVRFPNITNLCPFGEVFNASKFASVYAWNRKRISNCVADYSVLYNS-TSFSTFKCYGVSPTKLNDLCFTNVYADSFVVKGDEVRQIAPGQTGVIADYNYKLPDDF
SARS-CoV-2 RVQPTESIVRFPNITNLCPFGEVFNATRFASVYAWNRKRISNCVADYSVLYNS-ASFSTFKCYGVSPTKLNDLCFTNVYADSFVIRGDEVRQIAPGQTGKIADYNYKLPDDF
RaTG13     RVQPTDSIVRFPNITNLCPFGEVFNATTFASVYAWNRKRISNCVADYSVLYNS-TSFSTFKCYGVSPTKLNDLCFTNVYADSFVITGDEVRQIAPGQTGKIADYNYKLPDDF
Rs4081     RVSPTHEVVRFPNITNRCPFDKVFNASRFPNVYAWERTKISDCVADYTVLYNS-TSFSTFKCYGVSPSKLIDLCFTSVYADTFLIRSSEVRQVAPGETGVIADYNYKLPDDF
Yun11      RVSPSTEVIRFPNITNRCPFDRVFNASRFPSVYAWERTKISDCVADYTVLYNS-TSFSTFKCYGVSPSKLIDLCFTSVYADTFLIRFSEVRQIAPGETGVIADYNYKLPDEF
RmYN02     RILPSTEVVRFPNITNFCPFDKVFNATRFPNVYAWQRTKISDCIADYTVLYNS-TSFSTFKCYGVSPSKLIDLCFTSVYADTFLIRFSEVRQIAPGETGVIADYNYKLPDDF
Rf1        RVSPVTEVVRFPNITNLCPFDKVFNATRFPSVYAWERTKISDCVADYTVFYNS-TSFSTFNCYGVSPSKLIDLCFTSVYADTFLIRFSEVRQVAPGQTGVIADYNYKLPDDF
           *: *  .::******: ***..*** : * .****:* :*::*:***:*:*** : ****:***** :** ****:.**** *::  .:***:**.:** **********:*

           430       440       450       460       470       480       490       500       510       520       530       540
SARS       MGCVLAWNTRNIDATSTGNYNYKYRYLRHGKLRPFERDISNVPFSPDGKPCTP-PALNCYWPLNDYGFYTTTGIGYQPYRVVVLSFELLNAPATVCGPKLSTDLIKNQCVNF
WIV1       TGCVLAWNTRNIDATQTGNYNYKYRSLRHGKLRPFERDISNVPFSPDGKPCTP-PAFNCYWPLNDYGFYITNGIGYQPYRVVVLSFELLNAPATVCGPKLSTDLIKNQCVNF
SHC014     LGCVLAWNTNSKDSSTSGNYNYLYRWVRRSKLNPYERDLSNDIYSPGGQSCSA-VGPNCYNPLRPYGFFTTAGVGHQPYRVVVLSFELLNAPATVCGPKLSTDLIKNQCVNF
BM48-31    TGCVIAWNTNSLDS--SNE--FFYRRFRHGKIKPYGRDLSNVLFNPSGGTCSA-EGLNCYKPLASYGFTQSSGIGFQPYRVVVLSFELLNAPATVCGPKQSTELVKNKCVNF
BtKY72     TGCVLAWNTNSVDSKSGNN--FYYRLFRHGKIKPYERDISNVLYNSAGGTCSSISQLGCYEPLKSYGFTPTVGVGYQPYRVVVLSFELLNAPATVCGPKKSTELVKNKCVNF
pang17     TGCVIAWNSVKQDALTGGNYGYLYRLFRKSKLKPFERDISTEIYQAGSTPCNGQVGLNCYYPLERYGFHPTTGVNYQPFRVVVLSFELLNGPATVCGPKLSTTLVKDKCVNF
SARS-CoV-2 TGCVIAWNSNNLDSKVGGNYNYLYRLFRKSNLKPFERDISTEIYQAGSTPCNGVEGFNCYFPLQSYGFQPTNGVGYQPYRVVVLSFELLHAPATVCGPKKSTNLVKNKCVNF
RaTG13     TGCVIAWNSKHIDAKEGGNFNYLYRLFRKANLKPFERDISTEIYQAGSKPCNGQTGLNCYYPLYRYGFYPTDGVGHQPYRVVVLSFELLNAPATVCGPKKSTNLVKNKCVNF
Rs4081     TGCVIAWNTAKQDQG-----QYYYRSSRKTKLKPFERDLTSDE--------------NGVRTLSTYDFYPNVPIEYQATRVVVLSFELLNAPATVCGPKLSTALVKNQCVNF
Yun11      TGCVIAWNTANQDRG-----QYYYRSSRKTKLKPFERDLSSDE--------------NGVRTLSTYDFYPSVPLEYQATRVVVLSFELLNAPATVCGPKLSTSLIKNQCVNF
RmYN02     TGCVLAWNTAQQDIG-----SYFYRSHRAVKLKPFERDLSSDE--------------NGVRTLSTYDFNPNVPLDYQATRVVVLSFELLNAPATVCGPKLSTQLVKNRCVNF
Rf1        TGCVIAWNTAKQDVG-----SYFYRSHRSSKLKPFERDLSSEE--------------NGVRTLSTYDFNQNVPLEYQATRVVVLSFELLNAPATVCGPKLSTSLVKNQCVNF
            ***:***:   *        : **  *  ::.*: **::.              .    *  *.*  .  : .*  **********:.******** ** *:*::****
```

* = fully conserved
: = strongly similar
. = weakly similar

Binding site for EY6A: Conserved through panel
Mutated relative to SARS-CoV-2

Supplementary Fig. 9 EY6A binding to SARS-CoV-2 RBD. (**a**) EY6A Fab, shown in magenta, binding to SARS-CoV-2 RBD, shown in gray, with the contact site colored in blue. Based on PDB ID: 6ZER. (**b**) Binding interface of EY6A on SARS-CoV-2 RBD with the interaction residues shown in stick representation, based on PDB ID: 6ZER. Blue indicates residues at the binding site for EY6A conserved in all twelve sarbecovirus RBD constructs. Pink indicates a binding site residue in SARS-CoV-2 not conserved in other sarbecovirus RBD constructs. (**c**) Sarbecovirus RBD sequence alignment. Residues are colored as in (b). The amino acid numbering is based on the Spike protein of SARS-CoV-2.

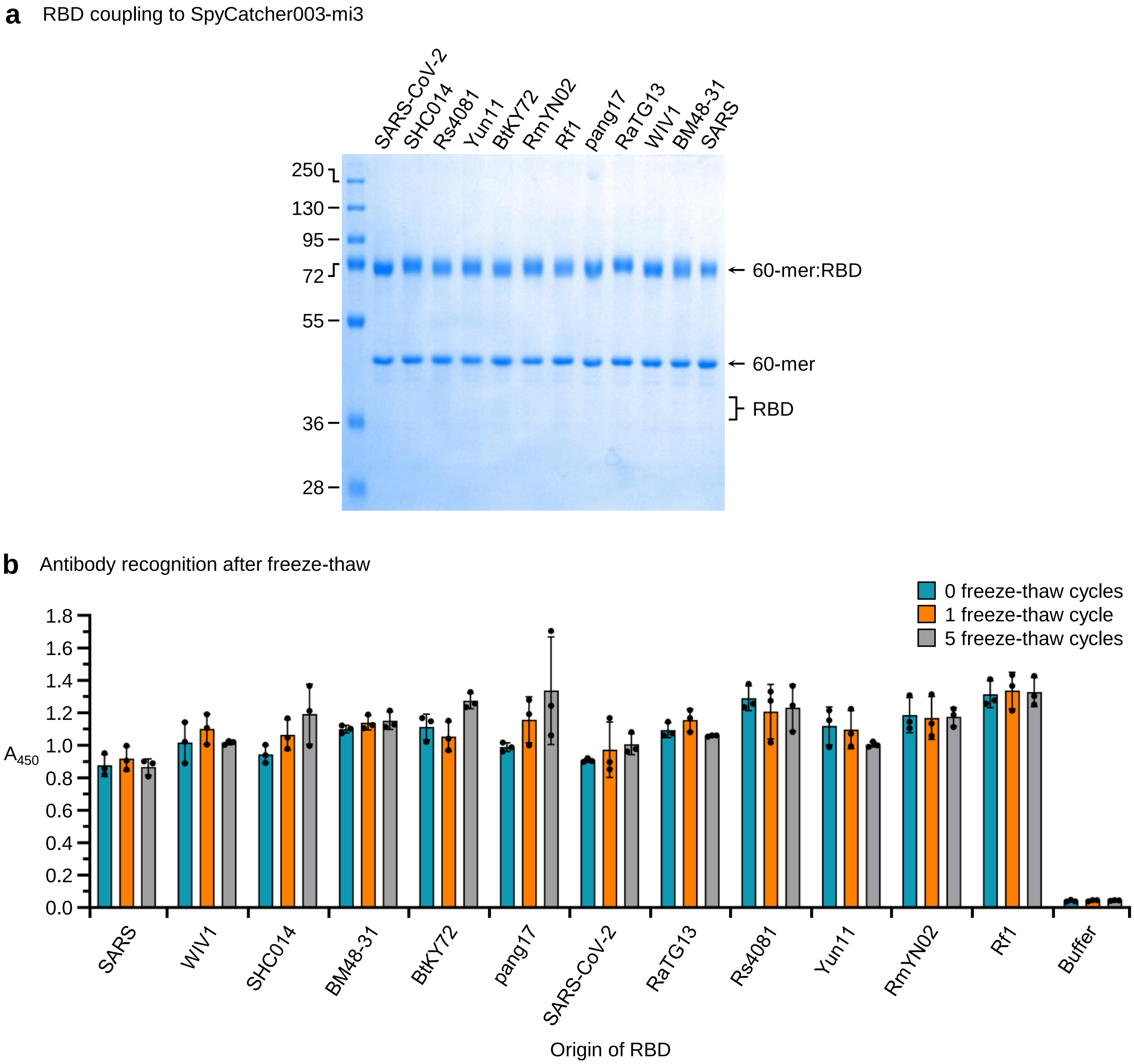

Supplementary Fig. 10 SpySwitch-purified RBDs are efficiently coupled to VLPs and resilient to freeze-thaw. (**a**) VLP coupling. Sarbecovirus RBD constructs were coupled to SpyCatcher003-mi3 by reacting 2 μM of each construct for 16 h at 4 °C in neutralized SpySwitch elution buffer. SDS-PAGE/Coomassie shown after coupling reaction. Molecular weight markers represent kDa. (**b**) Resilience to freeze-thaw. Recognition of sarbecovirus RBDs coupled to SpyCatcher003-mi3 by the conformation-sensitive antibody EY6A, after the coupled RBDs were subjected to the indicated number of freeze-thaw cycles. Individual data points and the mean absorbance were plotted, with error bars denoting ± 1 s.d. (n = 3). 0 freeze-thaw cycles in blue, 1 freeze-thaw cycle in orange and 5 freeze-thaw cycles in gray. Source data are provided as a Source data file.

a

Construct	Predicted pI
SpyTag003-β-galactosidase	5.5
SpyTag-MBP	5.7
SpyTag-sfGFP	6.3
SpyTag002-sfGFP	6.5
SpyTag003-sfGFP	6.7
H3/Vic-SpyTag	7.7
H3/Vic-SpyTag003	8.2
Rf1-CoV RBD-SpyTag003	8.6
BM48-31-CoV RBD-SpyTag003	8.7
RmYN02-CoV RBD-SpyTag003	8.8
Fab0.11-SpyTag	8.8
BtKY72-CoV RBD-SpyTag003	8.9
SARS-CoV RBD-SpyTag003	8.9
WIV1-CoV RBD-SpyTag003	8.9
Fab0.11-SpyTag002	8.9
Rs4081-CoV RBD-SpyTag003	8.9
Yun11-CoV RBD-SpyTag003	8.9
SHC014-CoV RBD-SpyTag003	8.9
Fab0.11-SpyTag003	9.0
RaTG13-CoV RBD-SpyTag003	9.1
pang17-CoV RBD-SpyTag003	9.1
SARS-CoV-2 RBD-SpyTag003	9.2

b SpySwitch pH elution of SpyTag-MBP from bacterial lysate

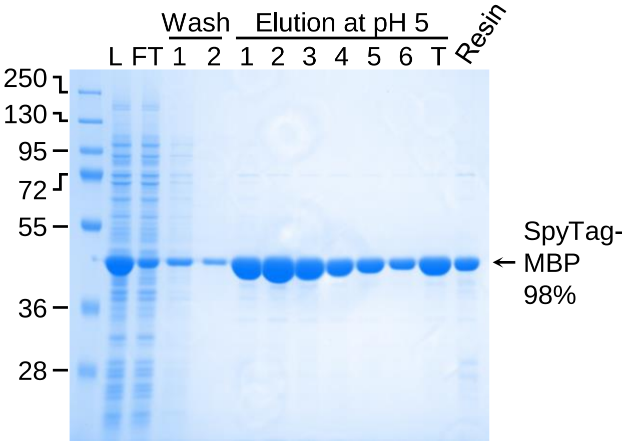

c SpySwitch pH elution of SpyTag003-β-galactosidase from bacterial lysate

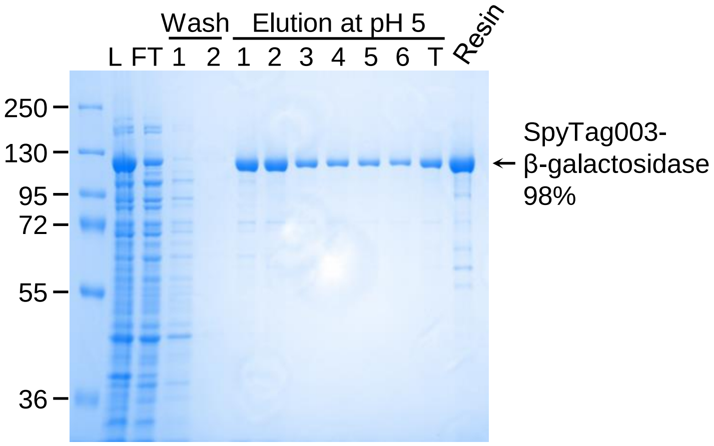

Supplementary Fig. 11 Proteins purified by SpySwitch. (**a**) Predicted isoelectric point (pI) of proteins purified by SpySwitch, based on ProtParam values calculated without glycosylation. (**b**) Purification of SpyTag-MBP by SpySwitch pH elution from bacterial lysate. (**c**) Purification of SpyTag003-β-galactosidase from bacterial lysate by SpySwitch pH elution. Samples were analyzed by reducing SDS-PAGE with Coomassie staining. % purity was calculated by densitometry. L, lysate expressing protein of interest; FT, flow-through; T, total pooled elution fractions; Resin, protein left on resin following elution. Molecular weight markers represent kDa. Source data are provided as a Source data file.

Construct	pH	ΔH (kcal/mol)	T_m (°C)	FWHM (°C)
SpySwitch	8	34.6	36.9	22.4
	7	28.7	41.8	20.9
	6	23.6	50.4	17.6
	5	14.4	57.7	17.7
SpyDock	8	15.2	50.1	23.3
	7	18.2	50.5	23.9
	6	28.0	53.4	21.5
	5	22.3	59.1	22.6

Supplementary Table 1 DSC parameters of SpySwitch and SpyDock. DSC of SpySwitch or SpyDock at pH 5.0, 6.0, 7.0 or 8.0 in SPG buffer. ΔH, T_m and FWHM (full width at half maximum) are given at each pH.

Construct	ΔH (kcal/mol)	T_m (°C)	FWHM (°C)
SARS	101	59.8	7.3
WIV1	104	57.5	8.5
SHC014	140	62.7	5.9
BM48-31	88.7	59.9	8.0
BtKY72	99.9	58.4	7.6
pang17	96.8	59.5	6.9
SARS-CoV-2	99.4	52.8	6.9
RaTG13	96.1	52.0	6.8
Rs4081	86.1	61.6	9.5
Yun11	83.5	63.5	9.8
RmYN02	81.4	62.5	10.3
Rf1	73.1	62.2	10.1

Supplementary Table 2 DSC parameters of sarbecovirus RBD constructs. DSC was performed in PBS pH 7.4. ΔH, T_m and FWHM (full width at half maximum) are given.

	Origin of RBD												
Antibody	SARS	WIV1	SHC014	BM48-31	BtKY72	pang17	SARS-CoV-2	RaTG13	Rs4081	Yun11	RmYN02	Rf1	No RBD
CR3022	1.077 ± 0.012	1.074 ± 0.023	1.161 ± 0.031	0.047 ± 0.007	1.05 ± 0.013	0.966 ± 0.066	1.07 ± 0.039	1.078 ± 0.021	1.061 ± 0.049	1.003 ± 0.031	1.023 ± 0.049	0.057 ± 0.022	0.05 ± 0.011
EY6A	1.145 ± 0.029	1.144 ± 0.013	1.113 ± 0.014	1.057 ± 0.04	1.048 ± 0.029	1.05 ± 0.033	1.076 ± 0.008	1.127 ± 0.01	1.104 ± 0.044	1.104 ± 0.044	1.072 ± 0.021	1.089 ± 0.044	0.044 ± 0.004
FP-12A	0.033 ± 0.004	0.051 ± 0.002	0.047 ± 0.004	0.041 ± 0.003	0.244 ± 0.01	0.191 ± 0.013	1.141 ± 0.076	0.161 ± 0.012	0.045 ± 0.008	0.042 ± 0.009	0.042 ± 0.001	0.046 ± 0.003	0.043 ± 0.008
FI-3A	0.04 ± 0.007	0.046 ± 0.006	0.056 ± 0.01	0.04 ± 0.005	0.044 ± 0.006	0.047 ± 0.009	0.975 ± 0.076	0.082 ± 0.01	0.042 ± 0.003	0.042 ± 0.006	0.043 ± 0.007	0.042 ± 0.005	0.034 ± 0.002
FP-8A	0.037 ± 0.005	0.053 ± 0.004	0.037 ± 0.004	0.05 ± 0.01	0.047 ± 0.007	0.032 ± 0.006	1.08 ± 0.102	0.057 ± 0.004	0.039 ± 0.003	0.036 ± 0.003	0.049 ± 0.009	0.041 ± 0.004	0.041 ± 0.002
FD-5D	0.034 ± 0.006	0.061 ± 0.015	0.061 ± 0.008	0.05 ± 0.002	0.051 ± 0.015	0.041 ± 0.005	1.213 ± 0.059	0.055 ± 0.011	0.064 ± 0.004	0.048 ± 0.006	0.062 ± 0.015	0.054 ± 0.004	0.038 ± 0.004
LCA60	0.067 ± 0.007	0.087 ± 0.015	0.08 ± 0.005	0.051 ± 0.008	0.091 ± 0.013	0.083 ± 0.006	0.092 ± 0.007	0.063 ± 0.016	0.047 ± 0.004	0.037 ± 0.007	0.056 ± 0.012	0.058 ± 0.021	0.041 ± 0.005
None	0.049 ± 0.022	0.046 ± 0.009	0.062 ± 0.008	0.034 ± 0.005	0.053 ± 0.012	0.032 ± 0.002	0.067 ± 0.012	0.049 ± 0.016	0.04 ± 0.014	0.034 ± 0.005	0.056 ± 0.005	0.032 ± 0.007	0.043 ± 0.003

Supplementary Table 3 Recognition of sarbecovirus RBDs by a panel of antibodies. Mean absorbance from ELISA ± 1 s.d. (n = 3), based on the data in Fig. 5f.
