# Supplementary material for: SpySwitch enables pH- or heat-responsive capture and release for plug-and-display nanoassembly
Source: Nat Commun. 2022 Jun 28;13:3714. doi: 10.1038/s41467-022-31193-8 (PMC9240080; doi:10.1038/s41467-022-31193-8)
Supplement: Supplementary file 2 — Reporting Summary [file 41467_2022_31193_MOESM2_ESM.pdf]

nature portfolio

Corresponding author(s): Mark Howarth

Last updated by author(s): Jun 21, 2022

Reporting Summary

Nature Portfolio wishes to improve the reproducibility of the work that we publish. This form provides structure for consistency and transparency in reporting. For further information on Nature Portfolio policies, see our Editorial Policies and the Editorial Policy Checklist.

Statistics

For all statistical analyses, confirm that the following items are present in the figure legend, table legend, main text, or Methods section.

n/a	Confirmed	
☐	☒	The exact sample size (*n*) for each experimental group/condition, given as a discrete number and unit of measurement
☐	☒	A statement on whether measurements were taken from distinct samples or whether the same sample was measured repeatedly
☒	☐	The statistical test(s) used AND whether they are one- or two-sided<br>*Only common tests should be described solely by name; describe more complex techniques in the Methods section.*
☒	☐	A description of all covariates tested
☒	☐	A description of any assumptions or corrections, such as tests of normality and adjustment for multiple comparisons
☐	☒	A full description of the statistical parameters including central tendency (e.g. means) or other basic estimates (e.g. regression coefficient) AND variation (e.g. standard deviation) or associated estimates of uncertainty (e.g. confidence intervals)
☒	☐	For null hypothesis testing, the test statistic (e.g. *F*, *t*, *r*) with confidence intervals, effect sizes, degrees of freedom and *P* value noted<br>*Give P values as exact values whenever suitable.*
☒	☐	For Bayesian analysis, information on the choice of priors and Markov chain Monte Carlo settings
☒	☐	For hierarchical and complex designs, identification of the appropriate level for tests and full reporting of outcomes
☒	☐	Estimates of effect sizes (e.g. Cohen's *d*, Pearson's *r*), indicating how they were calculated

Our web collection on statistics for biologists contains articles on many of the points above.

Software and code

Policy information about availability of computer code

Data collection	SDS-PAGE and Western blot images were acquired using ImageLab version 5.2.1. Protein concentrations were determined using NanoDrop One software version 1.4.2. sfGFP fluorescence data were collected by CLARIOstar version 5.20 RS or FLUOstar Omega version 5.10 R2. qPCR data were collected using MxPro qPCR software version 4.10. ITC data were collected by MicroCal PEAQ-ITC Software version 1.3. DSC data were collected using MicroCal PEAQ-DSC Measurement Software version 1.53. ELISA and BCA data were collected with FLUOstar Omega version 5.10 R2.
Data analysis	SDS-PAGE and Western blot images were analyzed in ImageLab version 3.0. sfGFP fluorescence data were analyzed with MARS Data Analysis Software 3.02 R2, Microsoft Excel 365 version 16.0.13801.21072, GraphPad Prism 9.3.1 and Origin2021b. qPCR data were analyzed using MxPro qPCR software version 4.10. Mass spectrometry data were analyzed in Mass Hunter Qualitative Analysis software B.07.00 and Microsoft Excel 365. ITC data were analyzed by MicroCal PEAQ-ITC Analysis Software version 1.1.0.1262. DSC data were analyzed with MicroCal PEAQ-DSC Analysis Software version 1.53 and Origin2021b. ELISA data were analyzed using MARS Data Analysis Software 3.02 R2, Microsoft Excel 365 and GraphPad Prism 9.3.1. BCA data were analyzed using MARS Data Analysis Software 3.02 R2 and Microsoft Excel 365. Amino acid sequences were analyzed using ExPASy ProtParam (Gasteiger et al 2015, The Proteomics Protocols Handbook; https://web.expasy.org/protparam/). Signal sequence cleavage was predicted by Signal P 6.0. Protein structures were visualized in PyMOL version 2.0.6. A phylogenetic tree was constructed using MEGA X v 11.0.8 software. A multiple sequence alignment was performed used Clustal Omega v 1.2.4.

For manuscripts utilizing custom algorithms or software that are central to the research but not yet described in published literature, software must be made available to editors and reviewers. We strongly encourage code deposition in a community repository (e.g. GitHub). See the Nature Portfolio guidelines for submitting code & software for further information.

Data

Policy information about availability of data

All manuscripts must include a data availability statement. This statement should provide the following information, where applicable:

- Accession codes, unique identifiers, or web links for publicly available datasets
- A description of any restrictions on data availability
- For clinical datasets or third party data, please ensure that the statement adheres to our policy

Amino acid sequences of SpyDock and SpySwitch are available in Supplementary Fig. 1. Sequences of other constructs are available in GenBank as described in the section Plasmids and cloning. Plasmids encoding pDEST14-SpyDock, pDEST14-SpySwitch, pET28a-SpyTag-MBP, pET28a-SpyTag-sfGFP, pET28a-SpyTag003-sfGFP, pET28a-SpyTag003-MBP, pET28a-AviTag-SpyTag003-MBP, pET28a-SpyCatcher003-mi3 and pDEST14-SpyCatcher002-oPent have been deposited in the Addgene repository (https://www.addgene.org/Mark_Howarth/). Further information and request for resources and reagents should be directed to and will be fulfilled by the lead contact, M.H. Source data are provided with this paper.

Field-specific reporting

Please select the one below that is the best fit for your research. If you are not sure, read the appropriate sections before making your selection.

[x] Life sciences [ ] Behavioural & social sciences [ ] Ecological, evolutionary & environmental sciences

For a reference copy of the document with all sections, see nature.com/documents/nr-reporting-summary-flat.pdf

Life sciences study design

All studies must disclose on these points even when the disclosure is negative.

Sample size	No statistical methods were used to predetermine samples sizes. Experiments conducted in duplicate or triplicate are indicated at the appropriate place. For qualitative assessment, this is sufficient.
Data exclusions	No data were excluded from analyses. For DSC data analyses, data ranges were equally adjusted for all samples in the MicroCal PEAQ-DSC Analysis Software.
Replication	Where conducted, all replications were successful. For representative SDS-PAGE (Fig. 3a-f, Fig. 4d, f, Fig. 5b, d, Supplementary Fig. 2b, Supplementary Fig. 3a, b, Supplementary Fig. 6 a, c, e, Supplementary Fig. 8 a, c, Supplementary Fig. 10a, Supplementary Figure 11b, c), observations were confirmed at least once with similar or identical conditions. Phage display without arabinose titration was validated by Western blot (Fig. 2b) at least once under similar conditions. Other SDS-PAGE and Western blot (Supplementary Fig. 3c, d, Supplementary Fig. 6b, d, Supplementary Fig. 8b, d) are each the results of a single experiment, which is sufficient based on repeats of different experimental approaches and tags/proteins. SpySwitch and SpyDock DSC results (Fig. 4a, b) were confirmed at least once with identical conditions. Assessment of RBD melting temperature (Fig. 5c, right panel) was performed in two separate experiments. The RBD ELISA (Fig. 5f) was performed in triplicate and confirmed at least once with identical conditions. Mass spectrometry results (Supplementary Fig. 4a) were confirmed once with similar conditions. ITC data were confirmed at least once with identical conditions (Supplementary Fig. 4b, c). SpySwitch capacity (Supplementary Fig. 7) was confirmed once under similar conditions.
Randomization	Randomization was not necessary or relevant to this study. No patient data or animal studies are contained within this manuscript.
Blinding	Blinding was not necessary or relevant to this study. No patient data or animal studies are contained within this manuscript.

Reporting for specific materials, systems and methods

We require information from authors about some types of materials, experimental systems and methods used in many studies. Here, indicate whether each material, system or method listed is relevant to your study. If you are not sure if a list item applies to your research, read the appropriate section before selecting a response.

Materials & experimental systems

n/a	Involved in the study	
[ ]	[x]	Antibodies
[ ]	[x]	Eukaryotic cell lines
[x]	[ ]	Palaeontology and archaeology
[x]	[ ]	Animals and other organisms
[x]	[ ]	Human research participants
[x]	[ ]	Clinical data
[x]	[ ]	Dual use research of concern

Methods

n/a	Involved in the study	
[x]	[ ]	ChIP-seq
[x]	[ ]	Flow cytometry
[x]	[ ]	MRI-based neuroimaging

Antibodies

Antibodies used	EY6A, FP-12A, FI-3A, FP-8A, FD-5D, LCA60 and CR3022 were produced in house (Huang et al 2021, PLoS Pathog; Huang et al 2022, Theranostics; Corti et al 2015, PNAS; ter Meulen et al 2006, PLoS Med)<br>mouse polyclonal anti-SpyCatcher serum (Bruun et al 2018, ACS Nano)<br>anti-HA tag antibody (Rockland Immunochemicals, 600-401-384, RRID:AB_217929)<br>anti-GFP antibody (Thermo Fisher, MA5-15256, clone GF28R, RRID:AB_10979281)<br>anti-rabbit IgG HRP (Thermo Fisher, 65-6120, RRID:AB_2533967)<br>anti-mouse IgG HRP (Sigma-Aldrich, A4416, RRID:AB_258167)<br>anti-human IgG HRP (Sigma-Aldrich, A8667, RRID:AB_258404)
Validation	Antibodies EY6A, FP-12A, FI-3A, FP-8A, FD-5D and CR3022 were produced in house and have been verified to bind to the receptor binding domain of SARS-CoV-2; for many of these interactions crystal structures are available. LCA60 was produced in house and has been shown to bind the spike glycoprotein of MERS-CoV (Corti et al 2015, PNAS). Part of this study aimed to investigate binding of these antibodies to receptor binding domains of different sarbecoviruses.<br><br>Mouse polyclonal anti-SpyCatcher serum (Bruun et al 2018, ACS Nano) has been shown to bind to SpyCatcher and derivates thereof, including in this publication.<br><br>The anti-HA tag antibody was from Rockland Immunochemicals. This antibody recognizes the commonly used HA epitope tag, which can be fused to the N- or C-terminus of proteins of interest. The manufacturer has tested this antibody by ELISA, immunohistochemistry, and Western blotting, both against the peptide used for immunization, as well as against recombinant proteins containing an HA tag. The manufacturer further states that this antibody has reacted with all HA-tagged proteins tested, and that this antibody does not cross-react with endogenous proteins, as assessed by Western blotting of bacterial cell lysate. This antibody has been cited by more than 50 publications to date.<br><br>The anti-GFP antibody was from Thermo Fisher, clone GF28R. This antibody recognizes both native and denatured forms of green fluorescent protein (GFP) and its many derivates, including superfolder GFP. The antibody has successfully been used for applications including immunoprecipitation, ELISA, Western blotting, and immunofluorescence. This antibody has been verified to bind to the antigen stated and been cited in over 50 publications for its use in Western blotting.

Eukaryotic cell lines

Policy information about cell lines

Cell line source(s)	Expi293F cells (Thermo Fisher, RRID:CVCL_D615, A14635), ExpiCHO-S cells (Thermo Fisher, RRID:CVCL_5J31, A29133)
Authentication	No authentication of Expi293F and ExpiCHO-S cell was performed after cell line acquisition.
Mycoplasma contamination	Expi293F cells and original stocks of ExpiCHO-S cells were tested negative for mycoplasma contamination.
Commonly misidentified lines<br>(See ICLAC register)	No commonly misidentified cell line was used in this study.
